# Bio-physical characterisation of polynyas as a key foraging habitat for juvenile male southern elephant seals (*Mirounga leonina*) in Prydz Bay, East Antarctica

**DOI:** 10.1371/journal.pone.0184536

**Published:** 2017-09-13

**Authors:** Veda Malpress, Sophie Bestley, Stuart Corney, Dirk Welsford, Sara Labrousse, Michael Sumner, Mark Hindell

**Affiliations:** 1 Institute for Marine and Antarctic Studies, University of Tasmania, Hobart, Tasmania, Australia; 2 Antarctic Climate & Ecosystem Cooperative Research Centre, University of Tasmania, Hobart, Tasmania, Australia; 3 Australian Antarctic Division, Department of Environment, Kingston, Tasmania, Australia; 4 Sorbonne Universités, UPMC University, Paris 06, UMR 7159 CNRS-IRD-MNHN, LOCEAN-IPSL, Paris, France; Hawaii Pacific University, UNITED STATES

## Abstract

Antarctic coastal polynyas are persistent open water areas in the sea ice zone, and regions of high biological productivity thought to be important foraging habitat for marine predators. This study quantified southern elephant seal (*Mirounga leonina)* habitat use within and around the polynyas of the Prydz Bay region (63°E– 88°E) in East Antarctica, and examined the bio-physical characteristics structuring polynyas as foraging habitat. Output from a climatological regional ocean model was used to provide context for *in situ* temperature-salinity vertical profiles collected by tagged elephant seals and to characterise the physical properties structuring polynyas. Biological properties were explored using remotely-sensed surface chlorophyll (Chl-*a*) and, qualitatively, historical fish assemblage data. Spatially gridded residence time of seals was examined in relation to habitat characteristics using generalized additive mixed models. The results showed clear polynya usage during early autumn and increasingly concentrated usage during early winter. Bathymetry, Chl-*a*, surface net heat flux (representing polynya location), and bottom temperature were identified as significant bio-physical predictors of the spatio-temporal habitat usage. The findings from this study confirm that the most important marine habitats for juvenile male southern elephant seals within Prydz Bay region are polynyas. A hypothesis exists regarding the seasonal evolution of primary productivity, coupling from surface to subsurface productivity and supporting elevated rates of secondary production in the upper water column during summer-autumn. An advancement to this hypothesis is proposed here, whereby this bio-physical coupling is likely to extend throughout the water column as it becomes fully convected during autumn-winter, to also promote pelagic-benthic linkages important for benthic foraging within polynyas.

## Introduction

Antarctic coastal polynyas are areas of reduced sea ice cover within the coastal sea ice zone, largely maintained by offshore winds and oceanic currents advecting ice away from the coast [1]. Although constituting a relatively small area of the Southern Ocean (~ 1% of maximum sea ice area), coastal polynyas are responsible for an estimated 10% of sea ice production. The brine rejection as a result of ice formation can lead to the formation of dense shelf water on the continental shelf [2–4]. In certain areas, this may flow off-shelf to form Antarctic Bottom Water (AABW). AABW formation is one important process driving the global thermohaline (overturning) circulation and acts as a sink for both heat and CO_2_ [5, 6].

Due to the ice-free environment, particularly in early spring when solar radiation rapidly increases, polynyas are regions of enhanced oceanic primary and secondary production relative to surrounding habitat [7]. Consequently, polynyas also support relatively high densities of upper trophic level organisms [8]. The importance of sea-ice zones and in particular polynya regions for successful foraging of several significant Antarctic predators, such as whales [9], Antarctic fur and Weddell seals and seabird communities (especially Adelie penguins) [10–12], is increasingly well documented [13–18]. To better understand why polynyas are important to top predators requires some understanding of the processes operating within polynyas that lead to the concentration and/or increase in food availability.

A major constraint to polynya research has been the difficulty in observing water properties under the ice-covered regions. This is due to a combination of a lack of access by ships for much of the year [5], expense and logistical difficulty in deployment and recovery of mooring arrays [19] and the limited ability of satellites to remotely sense the water surface properties when it is covered by ice [20] and/or cloud. Investigating circulation processes is possible through the development of high-resolution ocean models such as the Regional Ocean Modelling System (ROMS) [21–24]; see also the user community guide (https://www.myroms.org/). However, *in situ* observations are essential for verifying and constraining circulation models.

Marine predators equipped with oceanographic sensors provide a solution to the lack of *in situ* observations, providing information on ocean structure and water mass processes in regions and seasons rarely observed with traditional oceanographic platforms [20, 25]. Southern elephant seals (*Mirounga leonina*, or SES) are far-ranging, deep-diving predators that regularly spend time within the sea ice environment and high-latitude waters during their lengthy post-moult foraging trips [26–28]. Conductivity-Temperature-Depth Satellite Relay Data Loggers (CTD SRDLs) are used to simultaneously record animal location, dive behaviour and hydrographic profiles [20]. The data can provide insight into animal behaviour (e.g. [27, 28]) as well as *in situ* environmental information (e.g. [5, 13, 20, 29]) over extended timescales.

Such tagging studies have significantly increased understanding of the use of oceanographic features by foraging seals, showing SES widely exploit oceanic frontal systems, the marginal and pack-ice and coastal shelf regions, and can display both pelagic and benthic diving behaviour [27, 28, 30–34]. During the post-moult migrations some individuals of the Kerguelen and Macquarie Island populations forage along the East Antarctic shelf region [5, 20, 27, 35], and various foraging indices (*e*.*g*. body condition, patch quality, prey encounter events) indicate this may comprise the most lucrative foraging habitat [27, 35, 36].

**Fig 1:**
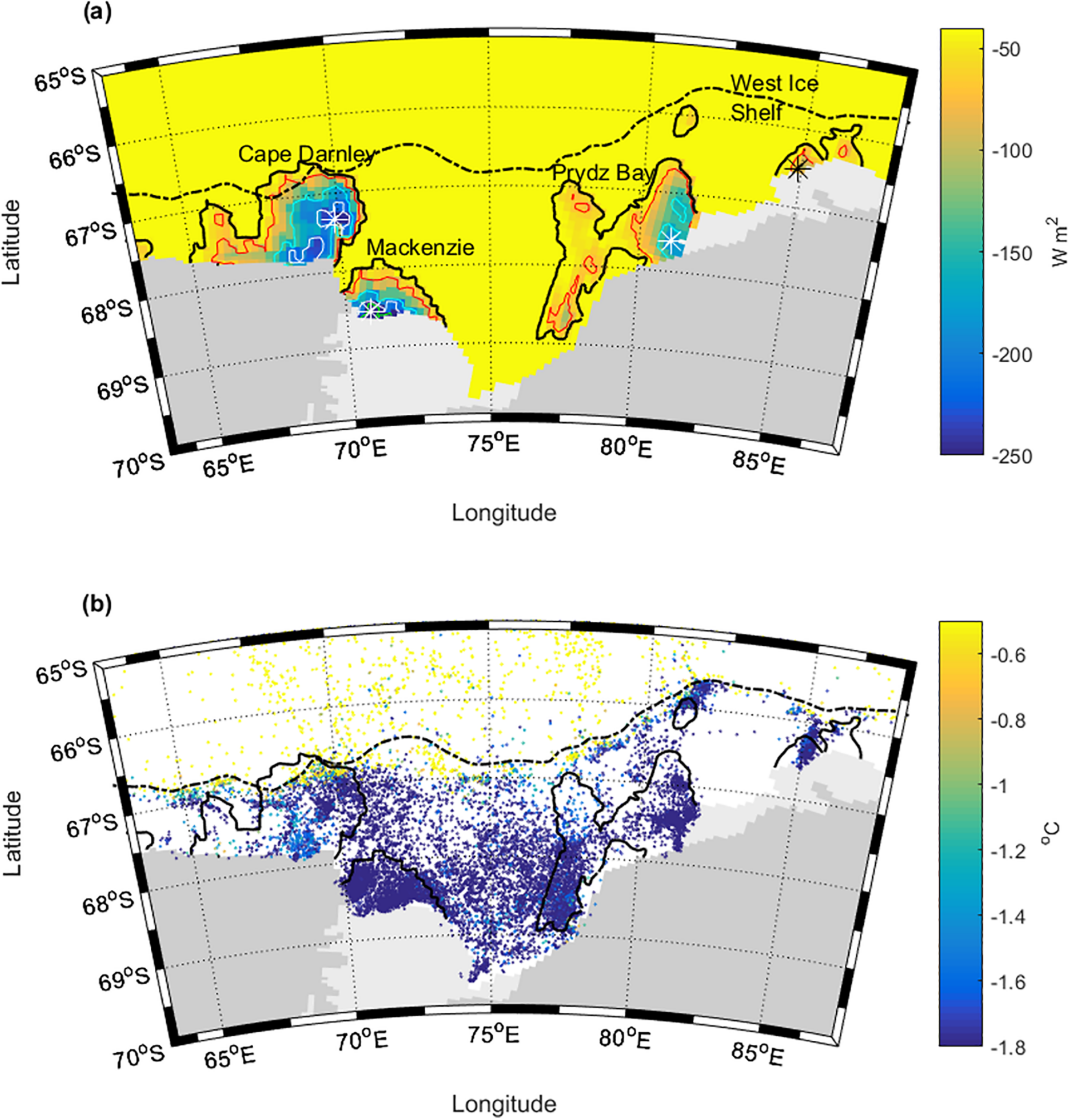
Map of the Prydz Bay study region. a) ROMS mean surface heat flux (expressed as W m^-2^) during the freezing season (March to October) and b) instrumented southern elephant seal CTD cast locations from the MEOP portal, 2007–2015 where data points are coloured by the deepest temperature readings per cast. In panel a) Heat flux contours of -40 W m^-2^ (black), -70 W m^-2^ (red), -150 W m^-2^ (cyan), -210 W m^-2^ (white) and -260 W m^-2^ (green) are shown. Polynyas from west to east are Cape Darnley (70°E), Mackenzie (72°E), Prydz Bay (82°E) and West Ice shelf (85°E). Centroid locations are indicated by a white or black star (Fig 1A). The Antarctic shelf break is represented by a black dashed line.

While studies of SES foraging behaviour have identified the importance of on-shelf regions in East Antarctica, the importance of specific habitat features within the region, such as polynyas, and the properties structuring these, have not been fully explored. This study aims to provide a bio-physical characterisation of polynyas as foraging habitat for SES specifically within the greater Prydz Bay region (63°E– 88°E) (Fig 1). Here, four coastal polynyas (Cape Darnley, Mackenzie, Prydz Bay and West Ice Shelf) play an important role in the sea-ice cycle [29], with the Cape Darnley polynya responsible for the second highest rate of polynya sea-ice production around Antarctica [1]. Additionally, this region is characterised by high rates of primary productivity [7] and significant benthic diversity [37].

The *in situ* CTD data collected from tagged seals provide invaluable observations of ocean properties; however, these can only describe the water characteristics in locations where SES were present. Model output from a climatological realisation of ROMS was used to provide a more complete spatial context for the region [21, 22]. Spatial bio-physical predictor fields, from ROMS and additionally satellite chlorophyll (Chl-*a*) data are examined as explanatory variables for statistical models predicting seal residence time. The results are used to develop a general hypothesis regarding the underlying physical-biological and pelagic-benthic coupling that supports where and how foraging habitat for marine predators occurs.

## Methodology

### Data sources

The spatial extent of the greater Prydz Bay study region from 63°E to 88°E includes four significant polynyas: Cape Darnley, Mackenzie Bay, Prydz Bay and West Ice Shelf (nomenclature as per Arrigo and Dijken [7]). The northern boundary of the study region was set at 65°S, in order to include the shelf break (Fig 1A), while the southern boundary was the Antarctic coastline (including the Amery ice shelf).

Several different datasets were integrated in this study. A ROMS implementation [21] provided the regional oceanographic context for the *in situ* observations collected by instrumented seals, and the two were used in conjunction to investigate dynamics within the four polynyas of interest. Additional biological information was obtained from remotely-sensed surface Chl-*a* data and an historical fish trawl database [38]. The habitat usage of seals within the greater Prydz Bay region was summarised as gridded residence time, as calculated from complete telemetry tracks, and modelled in response to selected bio-physical predictor fields. These datasets and the approaches used are detailed below.

#### Configuration of the Regional Ocean Modelling System (ROMS)

An existing climatological run of ROMS using present conditions (1992–2008) was used to provide oceanographic context for seal habitat. This implementation was a circumpolar expansion of an existing model [21] with a northern boundary at 30° S; however, this study focused on model output within the Prydz Bay region described above. Model output was available on a daily time step at a horizontal grid resolution of 0.25° and included depth-structured physical variables such as temperature, salinity, horizontal and vertical velocities. Daily atmospheric forcing was from the NCEPII reanalysis [39], with the northern boundary condition sourced from the ECCO2 reanalysis [40, 41]. The model used a mean state for surface initial condition and analytical initial conditions at depth.

This ROMS implementation used prescribed climatological surface heat and salt fluxes, to simulate ice production and coverage. These prescribed fluxes were based on ice concentrations from a climatology derived model using Special Sensor Microwave Imager (SSM/I) observations [1, 21]. This method forced heat and salt into the top of the water column [1] to overcome the poor performance of most ocean models in representing polynya locations and circulation processes.

#### Seal Conductivity-Temperature-Depth (CTD) casts

No new data was collected for this study; a previously collected seal dataset was utilised instead. Animal handlings were performed in accordance with relevant guidelines and regulations, after approval by the University of Tasmania and Macquarie University's Animal Ethics Committees for Australian deployments and by the Institut Paul-Emile Victor (IPEV) Ethics Committee for French deployments. For complete tagging and handling information, refer to Roquet *et al*. [25]. In brief, the seals were chemically sedated [42], weighed and measured [43], and a CTD-SRDL-9000 (Conductivity-Temperature-Depth Satellite Relay Data Logger, Sea Mammal Research Unit, St Andrews, UK) attached to the hair on the seal's head [44]. The combined weight of the tag and glue did not exceed 0.5 kg *i*.*e*. 0.15% of the mean departure weight of the seals. There is confidence that the instruments did not affect seal at-sea behaviour given that the smallest instrumented seal weighed 169 kg, making the tag <0.3% of the seal’s weight. It has been demonstrated that seals carrying twice this load (instruments of up to 0.6% of their mass) were unaffected in either the short-term (growth rates) or the long-term (survival) [45].

CTD-SRDLs collect and summarise data and transmit via the ARGOS satellite system when animals surface. These CTD data have been described in detail elsewhere [25, 46, 47], but briefly every vertical profile consists of temperature and salinity measurements at 17 depths (inflection points) determined on-board by a broken stick algorithm [47]. The tag deployments were supported under the Australian Integrated Marine Observing System through which all data are made publicly available(www.imos.org.au; [48]). The post-processed CTD data [25, 49] was sourced from the Marine Mammals Exploring the Oceans Pole to Pole public portal (www.meop.net/database).

The Prydz Bay regional subset included 58 SES that visited the study region during 2007, 2009 and 2011–2015. This included both French and Australian deployments at Kerguelen Island (*n* = 16) and at Davis Station (*n* = 42), Antarctica. These comprised almost all juvenile/sub-adult males (and one female seal), so age and sex effects were not considered. For the purposes of this study, population level habitat selection was the focus. The dataset was collated across all years to enable comparison with the climatological ROMS output and focus upon seasonal trends. For this, four periods were defined based on the distinct stages in the annual cycle of elephant seals [35, 50]; post-breeding (PB, November–January), post-moult 1 (PM1, February–April), post-moult 2 (PM2, May–July) and post-moult 3 (PM3, August–October). Due to the data availability (Table 1) for the purposes of statistical analysis only PM1 and PM2 are included.

**Table 1:** SES data summaries per season.

	Season				
	PM1	PM2	PM3	PB	Entire Dataset
**Years Available**	2007, 2009, 2011–2015	2009, 2011–2013	2009, 2011–2013	2007, 2011–2015	2007, 2009, 2011–2015
**Number of seals**	57	29	11	9	58
**Number of CTD Casts**	9514	4185	1456	527	15682
**Number of KF Locations**	29936	10349	2302	410	42997

The years in which data was available and the number of seals are displayed, as well as the number of CTD casts and Kalman filtered (KF) track locations (see Methods). PM1 = Post-moult 1 (Feb–Apr), PM2 = Post-moult 2 (May–Jul), PM3 = Post-moult 3 (Aug–Oct) and PB = Post-breeding (Nov–Jan).

#### Remotely sensed surface chlorophyll (Chl-*a*)

To provide information about the biological characteristics of the study region, and in particular polynyas, surface Chl-*a* data was examined. Two climatological fields were constructed for the study domain from monthly 8km gridded SeaWiFS/MODIS remotely sensed images over the period November 1997 to October 2008 (http://oceandata.sci.gsfc.nasa.gov/SeaWiFS/Mapped/Monthly/9km/Chlor_a/) using the R (R core development team 2015) package *raadtools* [51]. The climatologies were defined based on the elephant seal seasons as described above. There is a time lag between the development of phytoplankton blooms and the energy transfer up through trophic levels to higher order predators. Modelled estimates of the time lag to increases in the concentration of zooplankton grazers are in the order of 15–20 days [52] to around 30 days [53, 54] and most likely <90 days [53]. From this, there is a further time lag for the development of other secondary production (*i*.*e*.an ecological community of krill and other crustaceans, small to large fishes and squids) that is directly preyed upon by seals. To allow time for energy transfer between trophic levels, the Chl-*a* average for the season *prior* to each of PM1 and PM2 was used. It is known that SeaWiFS/MODIS underestimates Chl-*a* for the Southern Ocean, however a corrected product [55] was not available for the full time span of this study. As there is no comparison between Southern Ocean values with other oceanic regions, the relative values provided by this dataset are considered suitable for this analysis.

#### Historical fish data

The available historical pelagic and benthic fish data [56] was collated from the demersal trawls (Otter and Beam) on two historical voyages, AAMBER1 (17/2–5/3 1987) and AAMBER2 (17/2–28/2 1991) [38]. This dataset was spatially patchy but used as a qualitative indicator of species richness (total number of species) and approximate fish biomass within the region. There was greater availability of fish length records than weights within the database, and given that these parameters are related, length (mm) was used as a mass proxy. Total lengths for pelagic and benthic species were summed and divided by trawl effort; trawl effort was calculated from Speed (kn) x Tow Duration (min)/60.

### ROMS characterisation of polynyas

An animation of ROMS daily surface temperatures, which shows activity especially within the Cape Darnley and Mackenzie polynyas, can be found in S1 Video.

#### Virtual moorings

Virtual moorings were used to ensure ROMS was adequately simulating oceanographic conditions, as well as to characterise each polynya’s seasonal trends. Contours of net surface heat flux during the freezing season, March–October [1] were used to define the broader polynya region (Fig 1A) and a small centroid area defined for finer scale investigation. Due to the differing polynyas sizes the Cape Darnley and Mackenzie polynya centroids were a 3 x 3 (~0.75° x 0.75°) grid cell area, whereas Prydz polynya was 2 x 2 (~0.5° x 0.5°) and the West Ice shelf 1 x 2 (~0.25° x 0.5°). It was ensured that the grid cells were neither bordering land nor ice shelves as a precaution to avoid artefacts on the environmental variables of focus. Oceanographic time-series were constructed from ocean properties averaged across cells within the centroid regions, within the top and bottom 50 m of the water column. Supplementary time-series showing full-year temperatures and salinities at depth can be found in S1 Appendix.

#### Temperature-Salinity plots

Temperature-Salinity (T-S) plots are oceanographic tools used to represent the physical properties of water masses. Such plots can provide insights on how the water column develops throughout a season. T-S plots were created to compare ROMS output with seal CTD data. For each polynya, all unique seal CTD casts were extracted from a heat flux contour larger than the centroid region (see S2 Appendix; due to the differing activity intensities these thresholds differed: Cape Darnley = -150 W m^-2^, Mackenzie = -210 W m^-2^, Prydz Bay = -110 W m^-2^, West Ice Shelf = -60 W m^-2^) and combined for all years to display seasonal changes in the water column. Within each polynya, a ROMS T-S profile was extracted for each grid cell within the centroid (e.g. 9 for Cape Darnley), over the time period equivalent to that represented by the SES data. The larger area of the contour was used to capture the SES data (see S2 Appendix), rather than the smaller ROMS centroid area, to account for potential error in position and to give a broader representation of polynya processes. A potential density surface (σ_2_ = 37.16 kg m^-3^) was used to approximate the neutral density of AABW (γ_*n*_ = 28.27) [2] and plotted together with the approximate freezing point of sea-water (-1.85°C) on all T-S plots.

#### Virtual transects

Spatial transects were constructed to further explore oceanographic conditions and seal distribution in and around polynyas. A transect running north-south from each polynya centroid was defined, ensuring the origin was at least two grid cells north of any land or ice shelves, and extending north past the shelf break. Each transect was 3 grid cells wide, approximating a width of 0.5° +/- 0.2°.

Temperature and salinity were averaged throughout the freezing period (March–October) and across longitude, but resolved vertically through the water column. The total number of individual seals and unique CTD casts were calculated per 0.25° grid cell along each transect to provide a visual representation of seal density and the quantity of available data in relation to the transect features. Full-year time-series animations of temperature along each polynya transect can be found in S2–S5 Videos.

### Characterisation of SES habitat use

#### Spatial residence time

The ARGOS tracks for all SES (*n* = 58) were filtered using a Kalman filter (KF) [57] to minimise positional errors and to estimate location points along movement paths at regular 2-hour intervals [35]. From this the average residence time (hours) within the study region was calculated for each seal and then averaged across all individuals, on a regular 0.25° x 0.25° longitude/latitude grid to match the ROMS resolution. This was calculated using the R package *trip* [58]. Results were calculated for both an annual average representation of time spent, and the two focal post-moult seasons (PM1 and PM2), and reprojected on to the ROMS grid for analyses. Since tracking data are presence-only, to make inference about habitat preference or selectivity would require some generation of pseudo-absences for habitat availability [59, 60] (e.g. via a Poisson point process or random track simulation). However, this process was considered overly sophisticated for the small spatial scale of this work. The entire modelled region is easily traversable by seals in a matter of days; and they could clearly cover this domain many times over during the post-moult period at sea of up to 8 months [25]. Rather than habitat preference or selectivity it is important to note only habitat usage was directly modelled here.

#### Statistical models

Habitat use (residence time) was modelled in response to a selected set of biophysical variables. Using the R package *mgcv* [61] initial models were tested fitting generalised additive models (GAMs) to the two seasonal residency datasets. The two final models were fitted using the *gamm*() function (for generalised additive mixed models, GAMMs) which interfaces to the R package *nlme* [62]. This enables the incorporation of a spatial correlation structure in the models to account for autocorrelation in the data [63].

#### Predictor variables

A total of 9 predictor variables were initially considered for each season, comprising 8 physical variables extracted from ROMS plus the remotely sensed surface Chl*-a*. These were: bathymetry, surface heat flux, surface temperature, bottom temperature, bottom velocity magnitude, the eastward (U), northward (V) and vertical (W) components of bottom velocity. Each of these was chosen because of their assumed relevance to structuring polynyas as foraging habitat.

The heat flux variable was averaged over the entire freezing period (March–October) to represent polynya location and intensity even post-activity *i*.*e*. during summer. This averaged heat flux was used to develop both PM1 and PM2 models. The magnitude of bottom velocity was calculated from √ (*u*^2^ + *v*^2^). As previously described, Chl-*a* predictor represented an average of the previous season to allow for a biological lag between primary and secondary production. To account for skewed distributions, Chl-*a* and bathymetry were log-transformed; the response variable (residence time) was also log-transformed (natural logarithm).

Variance inflation factors (VIFs) and correlation coefficients amongst predictor variables [64] were checked to reduce collinearity effects [65]. A fairly stringent threshold was employed, allowing a maximum VIF of 3 [64]; this reduced maximum correlation between variables to less than 0.8, which was considered reasonable (Figs B and D in S3 Appendix). Once the appropriate set of predictor variables had been identified (*i*.*e*. selected predictor variables had both correlation and VIFs lower than the defined thresholds) Akaike’s Information Criterion (AIC) [66] was used to build up models manually via a forward stepwise procedure (only complete observations were used; PM1 *n* = 3408; PM2 *n* = 2448). This process was chosen as addressing all possible combinations of terms would have been too computationally intensive. Furthermore, this method facilitated understanding of the contribution of individual terms. Initially, a generalised additive model (GAM) was fitted to each individual predictor and the variable with the smallest AIC value (indicating comparative model fit) selected as the first covariate. Further predictors were added until F-tests indicated non-significance (*i*.*e*. p > 0.05).

The final combination of predictors from each seasonal GAM was used to build a GAMM for each of the seasons PM1 and PM2. These GAMMs included a Gaussian correlation structure on latitude and longitude to address the spatial autocorrelation inherent within the data [67]. The complete statistical procedure and results, including the partial residual plots for the two final GAMMs, can be found in S3 Appendix. The fitted values from the final model for each season were mapped to show the predicted habitat usage across the greater Prydz Bay region.

## Results

The CTD casts and tracking locations from the 58 seals (Table 1) provided information across most years between 2007 and 2015 (Table 2) with most data (~90%) recorded during PM1 and PM2. This reflects the tendency for seals to arrive early in the year and stay in the shelf region for varying lengths of time (see also S2 Appendix). Overall the CTD dataset provided good spatial coverage of the study region, with observations across the shelf, within all four polynyas, and along the shelf break (Fig 1B).

**Table 2:** SES data summaries per polynya.

Polynya	Seals	Unique Casts	Total Weeks (Non-Continuous)	Total Year
**Cape Darnley**	20	817	36 (Jan–Nov)	2007, 2011–2015
**Mackenzie**	32	1649	18 (Feb–Sept)	2011–2013, 2015
**Prydz**	13	1642	33 (Mar–Nov)	2009, 2011–2012
**West Ice Shelf**	3	272	23 (May–Oct)	2013

### ROMS evaluation

During the freezing period (March–October, Fig 1A) the polynyas were clearly far more active, in terms of a much greater negative heat flux, than the surrounding ocean. Mackenzie and Cape Darnley had the two most active cores, with a peak heat flux of -260 W m^-2^ and -210 W m^-2^ respectively. For the weaker Prydz and West Ice Shelf polynyas, the maximum heat flux was -150 W m^-2^ and -70 W m^-2^ respectively. The polynya centroids were defined within these contours.

#### Seasonal temperature and salinity trends

**Fig 2:**
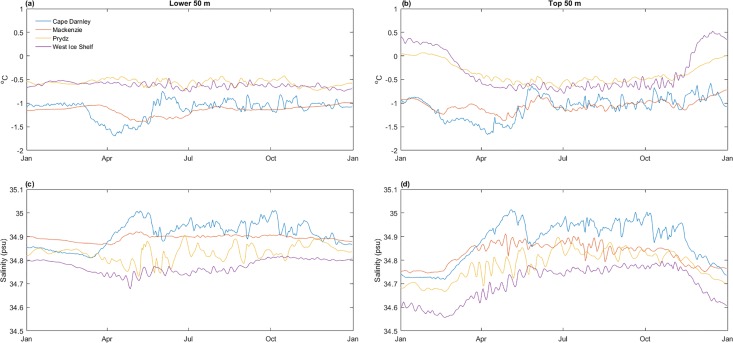
**Annual temperature (a) and (c) and salinity (b) and (d) time series from ROMS.** Temperature and salinity averaged over the centroid for each of the four polynyas, for approximately the lower 50m (LHS) and top 50m (RHS) of the water column, respectively. Bathymetry was extracted from the ROMS output. Legend for colours as shown in panel (a).

ROMS output for the four polynyas demonstrated a clear seasonal cycle of cooling and increasing salinity from the start of the freezing period (March-April), and the reverse in spring (mid-October; best seen in Fig 2D). Greater variability was evident in the top layer (Fig 2B and 2D) than the bottom layer (Fig 2A and 2C). The relationship between salinity and temperature was clear in both layers, with a temperature decrease corresponding to a (slightly lagged) salinity increase. This lag was most noticeable in Cape Darnley and Mackenzie leading into the freezing season, where surface and bottom temperatures were at a minimum. Cape Darnley polynya was the coldest and most saline polynya and showed the most variability within each month.

The ROMS time-series revealed unique signatures for each polynya (Fig 2 and S1 Appendix), but with similarities evident between the western (*i*.*e*. Cape Darnley and Mackenzie), and eastern (*i*.*e*. Prydz Bay and West Ice Shelf) pairs. Prydz Bay polynya and West Ice Shelf polynya were generally both warmer and fresher. While the seasonal and regional patterns were relatively well represented, in fact the ROMS representation of temperatures rarely approached the absolute freezing point of seawater (~ -1.85°C) in the western polynyas and not at all for the two easternmost polynyas; possibly in compensation to this the salinities were extremely high (e.g. commonly above 34.8 *psu*, Fig 2C and 2D).

#### Temperature-Salinity plots

**Fig 3:**
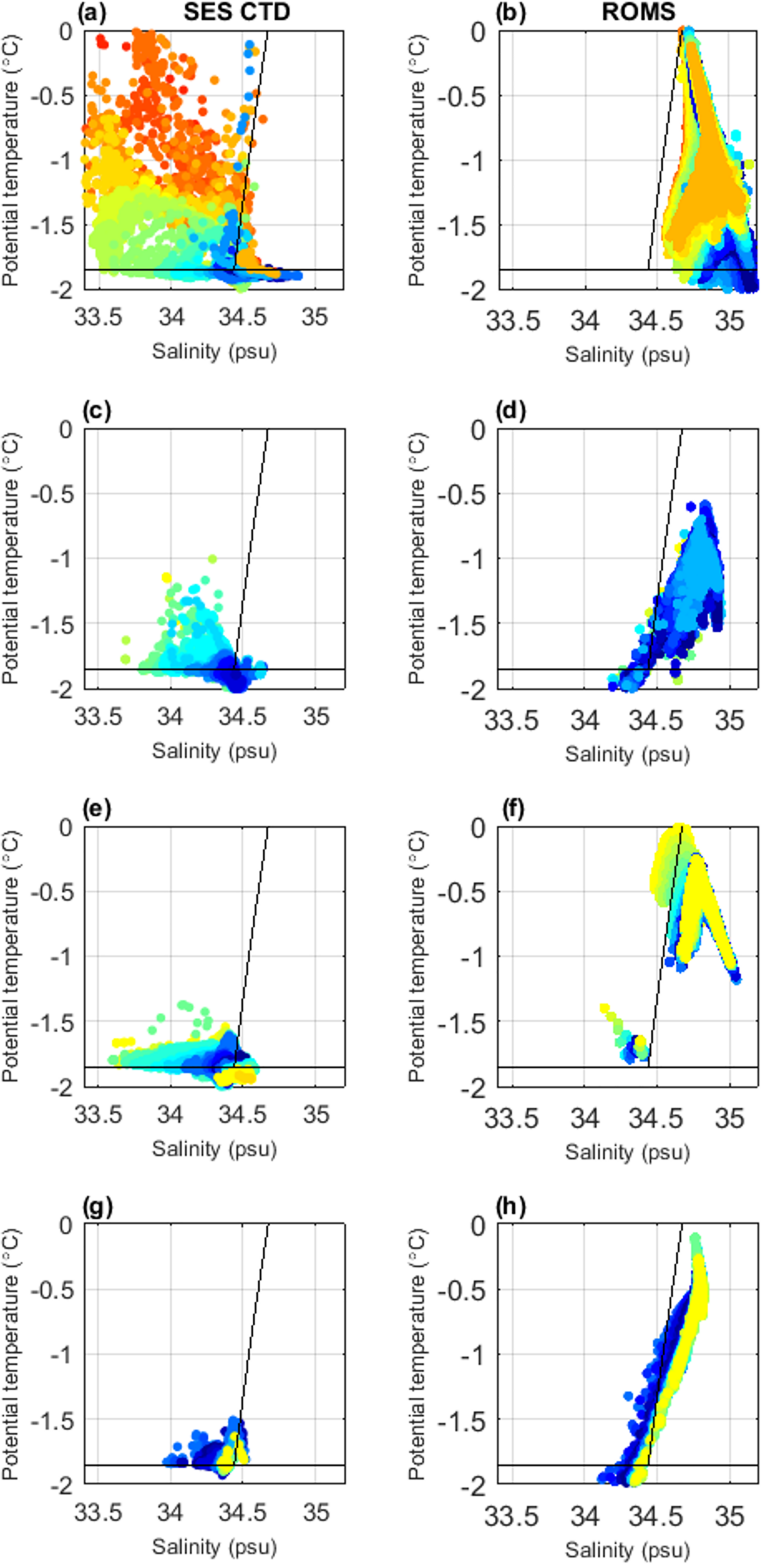
Comparative T-S plots between SES CTD (LHS) and ROMS (RHS) profiles within four East Antarctic polynyas. a, b) Cape Darnley (Jan–Nov), c, d) Mackenzie Bay (Feb–Sept), e, f) Prydz Bay (Mar–Nov) and g, h) West Ice Shelf (May–Oct). Profiles are coloured by day of year to show the seasonal progression, where summer is deep red leading into dark blue during the middle of winter. Seal data include all observations at all depths extracted from within a surface heat flux contour defining the most active region of each polynya (see S2 Appendix). The ROMS output displays a profile from every grid cell within the centroid, at fortnightly intervals. The approximate freezing point of water (-1.85°C) and the potential density curve representing AABW (σ_2_ = 37.16 kg m^-3^) are shown in black.

The T-S plots for each polynya (Fig 3) illustrated that the ROMS generated characteristics were more saline, by as much as 0.5–1 *psu*, than the *in situ* seal observations. More important than the absolute values from each data source is that the evolution of the water column from the seal observations (Fig 3A, 3C, 3E and 3G) was consistent with the evolution of water properties shown in the ROMS output (Fig 3B, 3D, 3F and 3H). Specifically, as the year progresses (plot points change from red, through orange, yellow, green then blue) the water gets colder and saltier. However, the seal observations were somewhat noisier than the ROMS output, which tended to occupy a smaller region of T-S space; this is particularly the case in summer (Fig 3A and 3B). This may be expected, simply due to the multi-year seal observations capturing more natural variability than a climatological model can produce. In general, AABW may potentially form when the cold, saline shelf waters reach sufficient density (*i*.*e*. potential density is greater than 37.16 kg m^-3^; the curve representing this density was shown as the diagonal line on all panels in Fig 3). ROMS represented most waters as sufficiently dense to be AABW precursor within all polynyas, and as such a realistic evaluation of this water mass formation was not possible. Due to the ROMS bias and difference in scale between the gridded ROMs output and seal observations, a one-to-one match between point values cannot be expected. Despite this bias, there was similarity in overall seasonal trends displayed between observed and modelled characteristics. For example, the water column structure at Cape Darnley from both observations and model output showed cooling throughout the season (Fig 3, panels a and b, light to dark blue), collapsing into a cold and highly saline water mass. For the other three polynyas, the seal observations throughout the autumn-winter were cold and saline while the ROMS representation was somewhat warmer.

#### Transects

**Fig 4:**
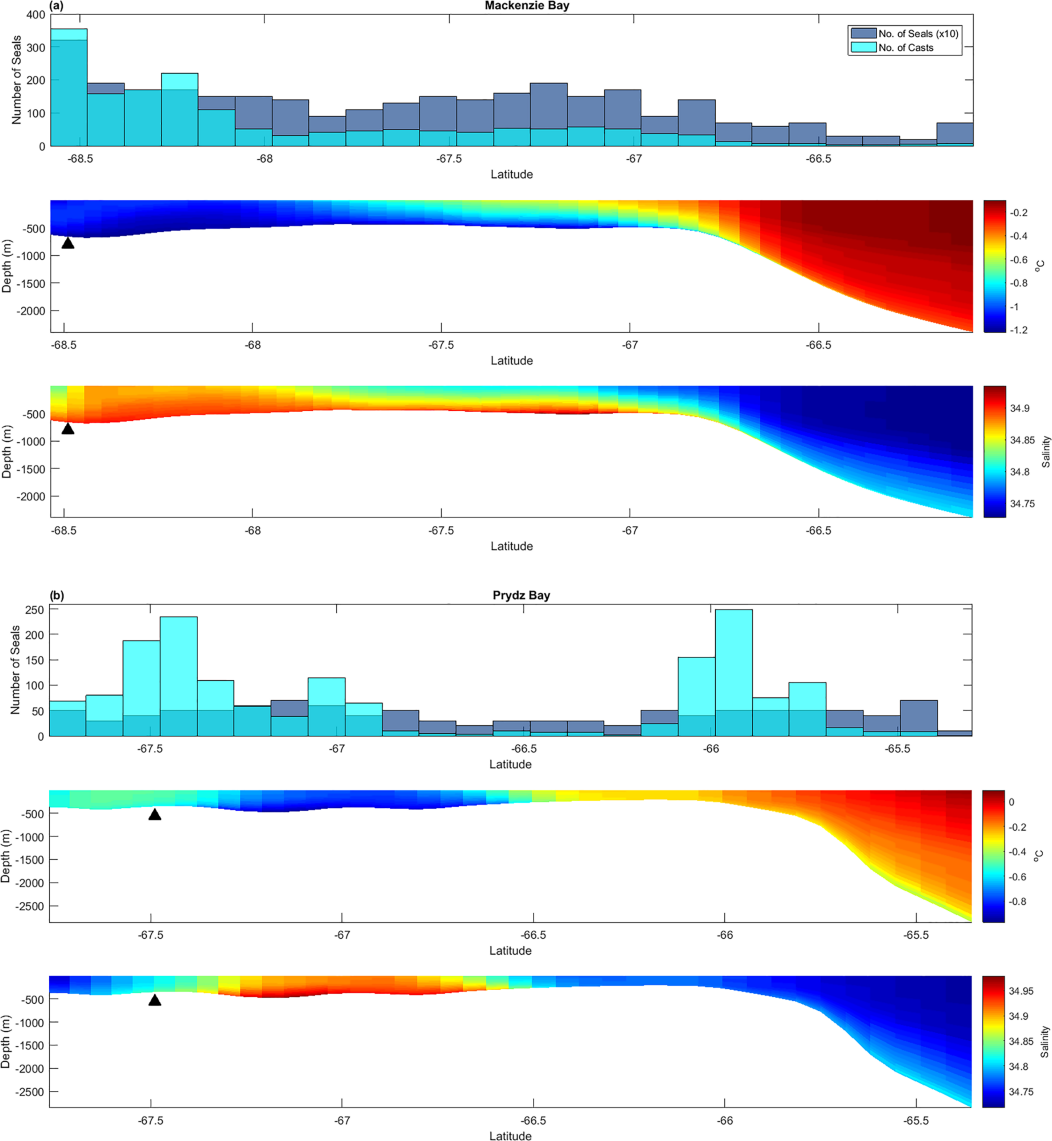
Virtual transects showing ROMS temperature and salinity in relation to the number of observed seals and seal CTD casts. Virtual transects ran north-south from polynya centres to the shelf break. Modelled temperature and salinity was averaged over the freezing period (March–October). Transects represent a) Mackenzie Bay and b) Prydz Bay polynyas. The other two polynya transects are available in S1 Appendix, Fig B. The number of seals (top panel) was multiplied by a factor (x10) for clarity. Centroid location is represented by a black triangle.

The virtual transects provided a spatial summary of ocean conditions and seal distribution within and around the four Prydz Bay polynyas (Fig 4 and time-series animations in S2–S5 Videos). The polynya centres were clearly apparent as areas of cold, saline water. There was some evidence of a downslope flow of cold, salty water from both Mackenzie (Fig 4A) and Cape Darnley polynyas (S1 Appendix), with potential off-shelf flow also in the vicinity of West Ice shelf polynya. The northern section of the transects approaching and crossing the shelf break, were dominated by a lens of warmer, fresher water. This lens overlaid the polynya water particularly in the vicinity north of Mackenzie, which represents the deepest and most southerly polynya.

For the Prydz Bay (Fig 4B) and West Ice Shelf polynyas, there was a concentration of seal CTD casts close to the Antarctic continent, with a greater number of tracked seals evident within the most active core areas (*e*.*g*. the cold, saline pocket around 67°S, Fig 4B). Along the Prydz Bay transect there was a second area of seal activity in a depression (~ 66°S) at the shelf break. The Cape Darnley transect also showed the greatest number of observations and seals not within the polynya core but closer to the shelf break (~ 66.9°S, S1 Appendix). Cape Darnley represents the shallowest polynya, with much of the shelf area being very cold and saline. For Mackenzie (Fig 4A) there was a high number of seals around both 68.5°S and 67°S, although the greatest number of observations were directly adjacent to the Amery Ice Shelf.

Taken together, the virtual moorings, consistent trends shown in the T-S plots and transect information indicated that the ROMS output for the polynyas was adequately reproducing seasonal water column trends. Furthermore, the differences between the modelled polynyas were sufficient to suggest that the model was satisfactorily capturing distinct regional behaviours.

### Characterisation of SES habitat use

#### Residence time results

**Fig 5:**
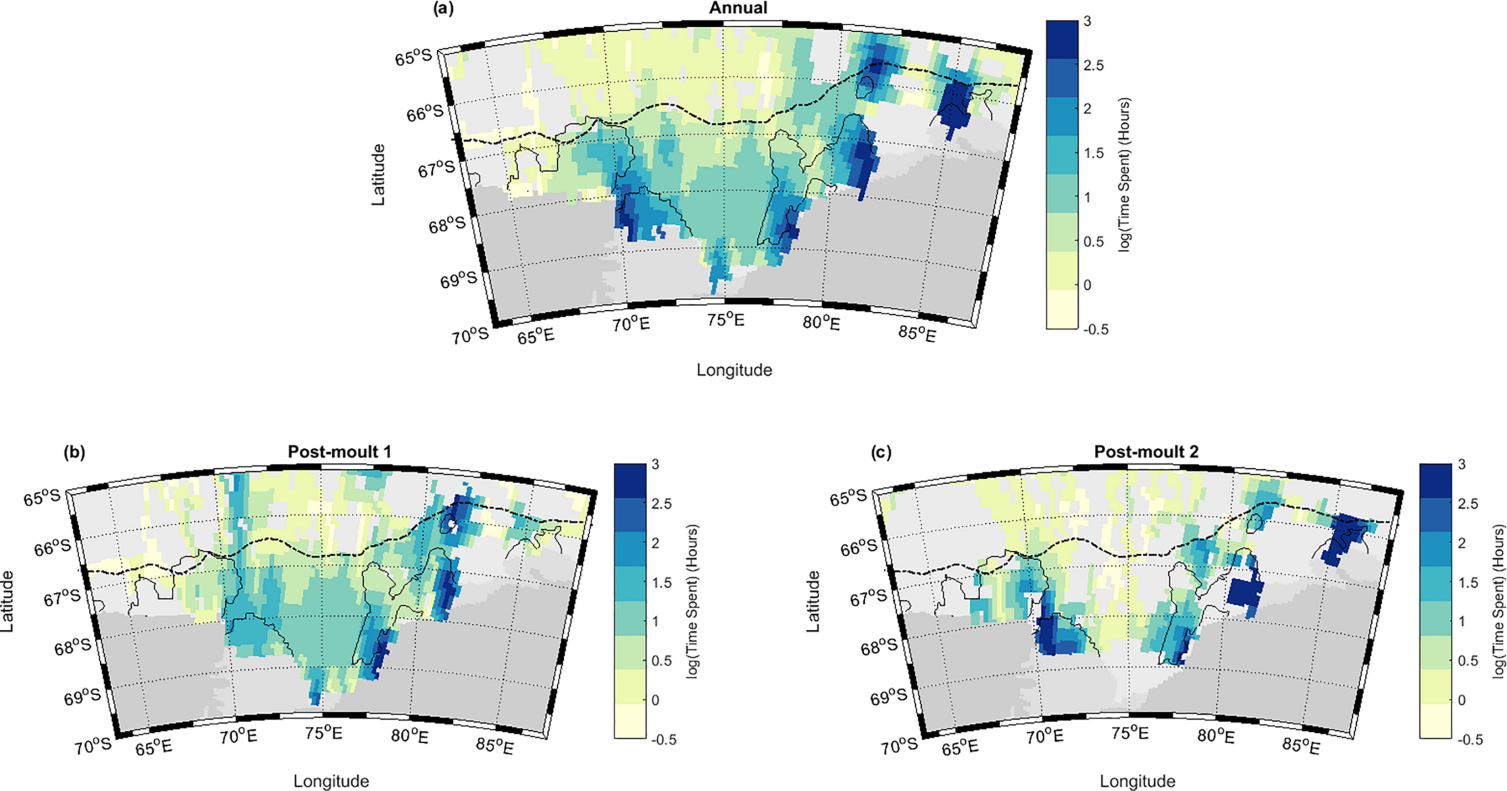
Maps showing the mean time spent per ROMS grid cell across all southern elephant seal individuals. Residence time represented a) annually and during b) Post-Moult 1 (PM1, February to April) and c) Post-Moult 2 (PM2, May to July). Greater Polynya regions are outlined with the -40 W m^-2^ heat flux threshold (black) and the 1500m isobath (dotted line) indicates the shelf break.

The spatially gridded time-spent data revealed habitat usage patterns strongly centred on polynyas (Fig 5). The annual summary clearly showed that of all the available foraging locations the greatest time was spent in the region of the four polynyas (Fig 5A). Although visited by a high number of individual seals (Table 2) the Cape Darnley polynya had less concentrated use (~8 hours maximum per grid cell) compared to the other 3 polynyas (~20 hours). Also apparent was a concentrated usage of the shelf break area north of the Prydz Bay polynya, as previously identified within the virtual transect.

During Post-moult 1 (*n* = 29936 KF locations, *N* = 57 seals) (Fig 5B) the highest residence time was in the Prydz Bay polynya and the shelf break area to the north (also indicated as a potentially active area by the -40 W m^-2^ contour). There was also evidence of a north-south transit route into the region, from a relatively concentrated usage observed along a route into Mackenzie Bay polynya near 71°E. For PM1 there was generally high usage across the entire shelf as compared with off-shelf, indicating that the entire area was largely accessible at this time. Though there was less data available during early winter (PM2, *n* = 10349, *N* = 29 seals) (Fig 5C), the spatial usage patterns showed a stronger contraction towards the polynya areas during sea-ice advance; West Ice Shelf, Prydz Bay and Mackenzie polynyas were all regions of concentrated time spent during PM2. While the concentration of seals in Cape Darnley was lower, there was still evidence of increased use in this polynya relative to the surrounding region.

#### Predictor fields

**Fig 6:**
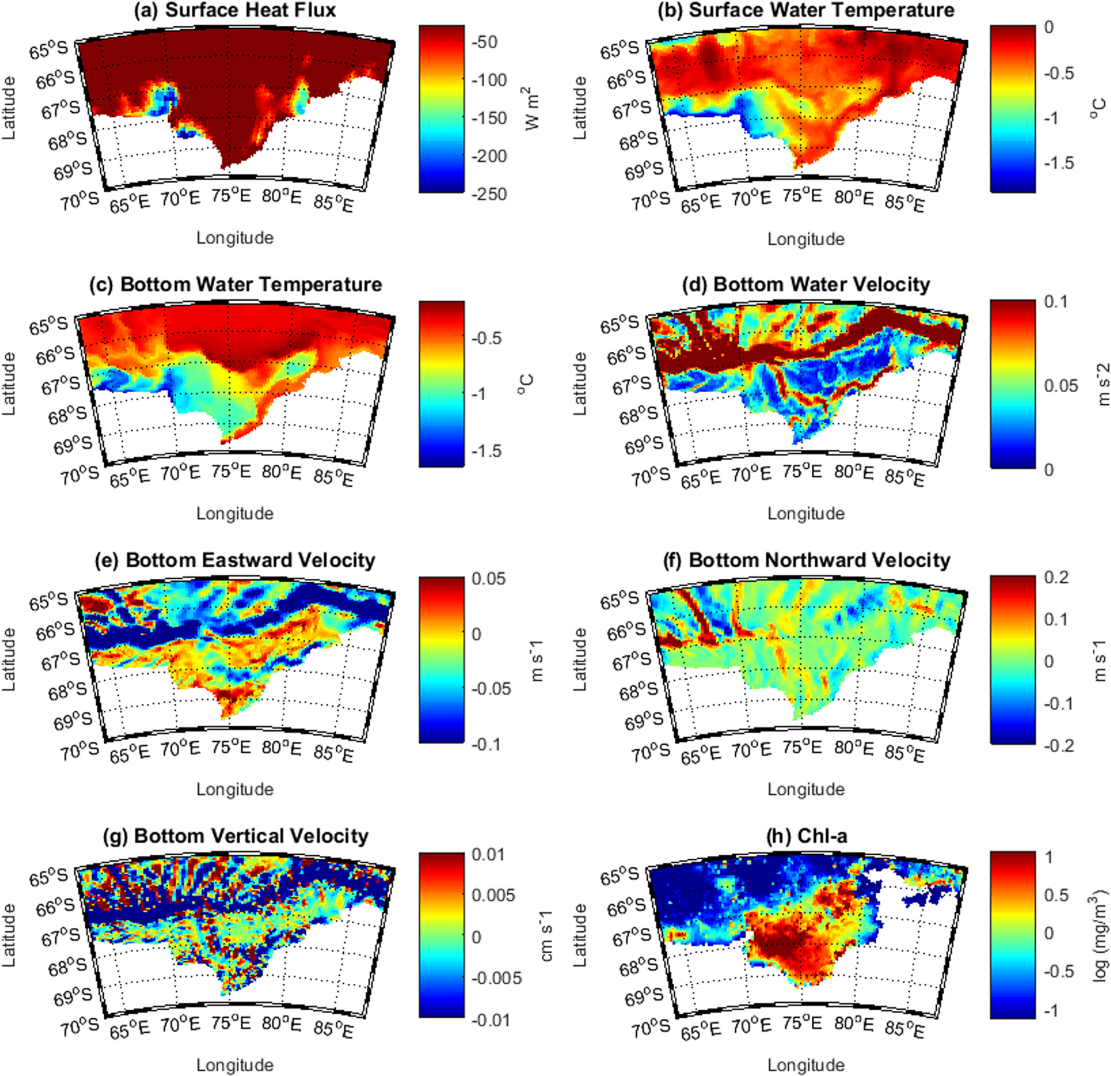
Physical and biological predictors fields used to build the seasonal GAMM for PM1. a) Polynya location, surface heat flux (W m-^2^) averaged over the freezing period (March to October); b) surface water temperature (°C); c) bottom temperature (°C); and the d) total magnitude (m s^-2^), e) eastward (m s^-1^) (U), f) northward (m s^-1^) (V), and g) vertical (cm s^-1^) (W) components of bottom water velocity; and h) log transformed surface Chl-*a* concentration (mg/m^3^) averaged over the previous season (November to January).

Due to the similarities between the predictor fields from each of the two seasons considered, only the fields for PM1 are shown (Fig 6). The predictor fields for PM2 are available in S3 Appendix.

ROMS surface water temperature for PM1 showed Cape Darnley and Mackenzie polynyas as distinctly colder than the surrounding region (Fig 6B). Bottom temperature (Fig 6C) additionally highlighted the cold core of Prydz Bay polynya. A warm on-shelf flow originating in the north-east of Prydz Bay near 84°E, and flowing westward was evident in the bottom temperature as well as the bottom velocity (Fig 6D) and eastward (U) velocity (Fig 6E) fields, revealing the cyclonic circulation in the middle of the bay. Additionally, there was evidence of a strong westward jet along the shelf break representing the Antarctic Slope Current. When examining northward velocity (V) (Fig 6F), off-shelf flows of cold water originating from Cape Darnley were evident. Surface Chl*-a* (Fig 6H) for the preceding spring season (*i*.*e*. November–January) showed highest concentrations in the middle of Prydz Bay, with elevated levels evident within Prydz Bay and Mackenzie polynyas.

#### Model predictions

**Fig 7:**
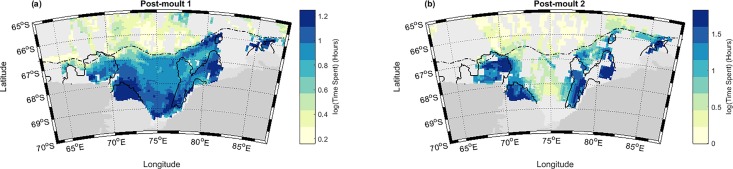
Generalised additive mixed model predictions of SES habitat selection. For (a) Post-Moult 1 (PM1) and (b) Post-Moult 2 (PM2). Grid cell resolution of 0.25°× 0.25°. For PM1, surface heat flux (W m-^2^), surface temperature (°C), bottom temperature (°C), current magnitude (m s^-2^) and northward (m s^-1^) velocities, log transformed bathymetry (m) and log transformed Chl-*a* (mg/m^3^) were retained. For PM2, surface heat flux (W m-^2^), bottom temperature (°C), current magnitude (m s^-2^), vertical (cm s^-1^) and northward (m s^-1^) water velocities, log transformed bathymetry (m) and log transformed Chl-*a* (mg/m^3^) were retained.

The goodness-of-fit statistics available for the full GAMs (PM1: adjusted R^2^ = 0.484, deviance explained = 49.2%; PM2: adjusted R^2^ = 0.589, deviance explained = 59.6%) and GAMMs (PM1: adjusted R^2^ = 0.415; PM2: adjusted R^2^ = 0.538) indicated a good fit from the final models, particularly given the complex spatial ecological data. The predictor variables reported as significant for the final PM1 and PM2 GAMMs are given in Table 3. The three most significant individual predictors for PM1 were: bathymetry (AIC = 4540.483, R^2^ = 0.284), Chl-*a* (AIC = 4969.957, R^2^ = 0.188) and bottom temperature (AIC = 5001.159, R^2^ = 0.181) (these cited values relate to single predictor models, see S3 Appendix). These three predictors were the primary focus, as the deviance explained dropped substantially for the rest of the variables. The U and W bottom velocities were not retained in the final GAMM for PM1 (Fig 7A), and surface temperature and U bottom velocity were not retained for PM2 (Fig 7B). The influence of bathymetry is clear in the generally increased time spent across the entire shelf region (Fig 7A); partial residual plots (S3 Appendix) revealed a preference for shelf depths (200–700 m), with lower residence time offshore. Bottom temperature, associated with surface heat flux (or polynya location), influenced the concentrated polynya usage; cold bottom temperatures were especially evident within the Mackenzie and Prydz Bay polynyas (Fig 6C). Increased residence time was associated with higher surface heat flux, and this predictor became more influential in PM2 (Figs C and E in S3 Appendix). Increasingly concentrated polynya usage was predicted for all four polynyas during PM2 relative to PM1 (Fig 7B).

**Table 3:** Statistical results from GAMMs fitted for (a) PM1 (residence time averaged over *n* = 57 seals, observations in *N* = 3408 grid cells), R^2^ (adjusted) = 0.415, and (b) PM2 (residence time averaged over *n* = 29 seals, observations in *N* = 2448 grid cells), R^2^ (adjusted) = 0.538.

	Estimated degrees of freedom	F value	p value
**(a)**			
**s(heat)**	4.690	15.456	<0.001 (***)
**s(s_temp)**	5.590	8.079	<0.001 (***)
**s(b_temp)**	5.070	5.295	<0.001 (***)
**s(vel)**	3.115	22.143	<0.001 (***)
**s(V)**	1.000	30.421	<0.001 (***)
**s(log.bath)**	8.661	35.041	<0.001 (***)
**s(log.Chlo)**	6.629	13.353	<0.001 (***)
**(b)**			
**s(heat)**	8.593	52.031	<0.001 (***)
**s(b_temp)**	8.433	15.618	<0.001 (***)
**s(vel)**	6.313	4.380	<0.001 (***)
**s(V)**	1.000	21.124	<0.001 (***)
**s(log.bath)**	7.347	27.868	<0.001 (***)
**s(log.Chlo)**	4.208	10.060	<0.001 (***)
**s(W)**	1.873	5.291	<0.01 (**)

Heat = net surface heat flux average over the freezing period (March–October); used to represent polynya location; all other were variables seasonally averaged: s_temp = surface temperature, b_temp = bottom temperature, vel = bottom velocity magnitude; V = northward and W = vertical components of bottom velocity; log.bath = log transformed bathymetry, log.Chlo = log transformed surface Chl-*a* (data from the previous season).

** = p value <0.01

*** = p value <0.001.

Statistical relationships for PM2 were similar to PM1, with heat flux followed in influence by bathymetry and Chl-*a* (Table D in S3 Appendix). In both seasons, the magnitude of currents also played an important role: habitat usage increased with lower levels of water movement, with seals spending relatively less time in the vicinity of higher speed flows along the shelf-break. Higher rates of downward vertical velocity along the shelf-break were weakly linked to an increase in predicted time spent (Figs D and E) in PM2.

**Fig 8:**
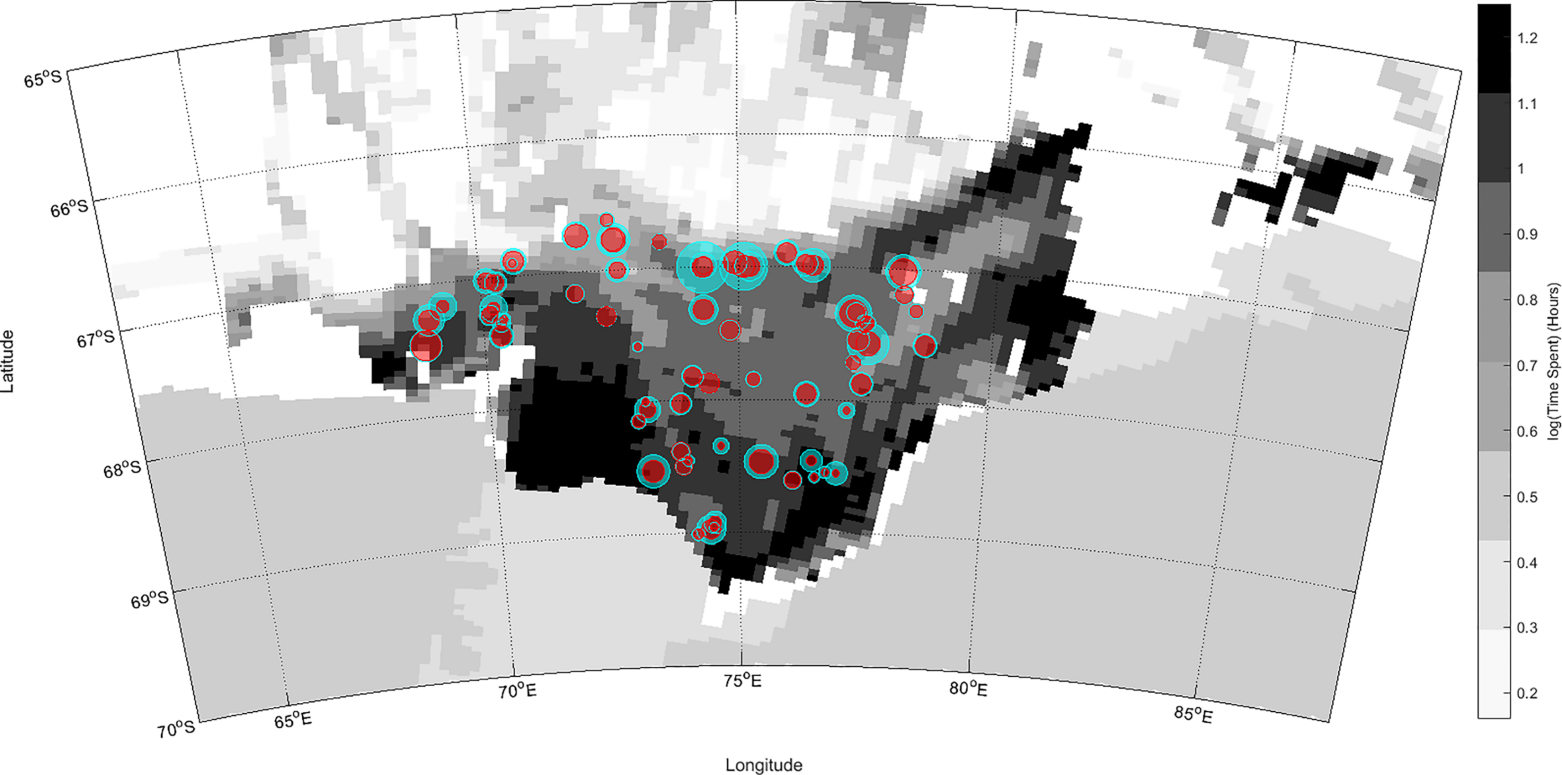
Historical pelagic and benthic fish data distribution, showing species richness (red) and proxy fish biomass (cyan). The size of the circle indicates the relative value of both indicators. Species richness is the total number of species, and the proxy biomass was obtained from the total summed fish length (mm) for pelagic and benthic species standardized by trawl effort (Speed [kn] x Tow Duration [min]/60, see Methods). Background shows predicted habitat selection for PM1.

The available historical fish data (Fig 8) had patchy spatial coverage, with trawls inside polynyas only occurring at Cape Darnley and around the boundary regions for the other three. Consequently, this dataset was only used to qualitatively examine spatial patterns. The greatest proxy fish biomass occurred around the shelf break and within the centre of the bay, with high biomass also apparent in the vicinity of the warm shelf inflow near 84°E. Trawls within the Cape Darnley polynya revealed a relatively abundant number of species as did one trawl immediately adjacent to the Amery ice shelf.

## Discussion

Southern elephant seals from breeding colonies in the South Indian Ocean (Kerguelen and Macquarie Islands) are known to forage around the Antarctic continental shelf and slope (e.g. [28, 30, 35, 36]) where they locate high-quality prey patches [36]. Characterising this key foraging habitat is not only ecologically important for our understanding of species responses to specific environmental conditions; the process also informs a more integrated understanding of these under-sampled regions. The physical importance of Antarctic coastal polynyas has been previously described (e.g. [1, 29]), and this study provides important new insights into the bio-physical properties structuring these as predator foraging habitat. This study clearly showed seals spending greater time on-shelf within the Prydz Bay vicinity in East Antarctica, and exhibiting concentrated residence times within the four coastal polynyas in the region. Statistical analyses relating a suite of bio-physical predictors showed an influence of bathymetry, Chl-*a*, surface heat flux, bottom temperature and velocity on seal residence time. This provides the first statistical description of polynya characteristics as a foraging habitat for southern elephant seals using an oceanographic model. A hypothesis has been previously proposed regarding surface-subsurface coupling of biological productivity in coastal polynyas [68]; an extension is proposed here to include pelagic-benthic coupling in the vicinity of coastal polynyas, which leads to favourable conditions in terms of resources for predators.

### Evaluation of ocean model output

The model evaluation process demonstrated that ROMS adequately represented the ocean properties and circulation in the study region for the purposes of this study. The ROMS output provided oceanographic context that supported two spatially correlated GAMMs with good fit to observed seal residence time enabling realistic predictions of habitat usage based upon bio-physical predictors.

#### Reproduction of main oceanographic features

Cape Darnley was the coldest and saltiest of the polynyas throughout all seasons, most likely a product of high rates of ice formation. Cape Darnley has been identified as having the second highest rate of ice production around Antarctica, behind the Ross Sea [1]. It is an important regional source of AABW, a cold dense water mass that is a major contributor to global overturning circulation [3, 5]. AABW originates as Dense Shelf Water formed through brine rejection during sea-ice production [2–4]. The formation of Dense Shelf Water begins in March, at the start of the freezing period [5]. ROMS model output (with the imposed surface heat and salt fluxes) demonstrated such a trend with an increase in salinity and a drop in temperature throughout the water column at the start of March. Additionally, downslope flows of Dense Shelf Water in a north-west direction from Cape Darnley during the freezing period have been described [5]. The ROMS bottom velocity components (U and V) showed some evidence of this outflow.

The Prydz Bay and West Ice Shelf polynyas exhibited warmer and less saline trends than Cape Darnley and Mackenzie. A large cyclonic gyre in the centre of Prydz Bay has been associated with a coastal current that circulates warm Modified Circumpolar Deep Water into the bay and across the calving front of Amery Ice Shelf (which forms the southern border of the bay) [21] and continues westward [69]. The various ROMS velocity components represented this flow, and the ROMS temperature time series for the Prydz Bay and West Ice Shelf polynyas reflected the influence of this warmer water.

The potential influence of the gyre and other circulation features such as eddies [70], or physical forcing from wind patterns [71] may explain the weekly cycles apparent in the temperature and salinity time series within both Prydz Bay and Mackenzie polynyas (Fig 2 and S1 Appendix). Additionally, the small T-S phase space occupied by the Mackenzie Bay polynya could be attributed to the accumulation of High Salinity Shelf Water due to the outflows from the Amery Ice Shelf. This cold, saline water mass, along with the isolation of Mackenzie due to surrounding bathymetry [29], may have contributed to the model simulating intensely cold, highly saline water throughout the year.

#### Model limitations

When comparing ROMS output to SES CTD profiles, a definite saline bias was evident in the modelled output. One likely cause is resolution of the model. The circumpolar domain of the model meant that the horizontal grid resolution was configured at 0.25°. This is at the coarse end of a ‘high’ resolution regional model and it is possible this was not adequate for simulating the fine scale processes within the region. In particular the model struggled to represent water properties as the column approached the freezing point, overcompensating regarding salinity [21, 72]. While it is known that CTD tags experience a shift in salinity due to the effect of the animal on conductance, the correction applied to the dataset [25] eliminates this potential issue. Furthermore, the seal observations were consistent with recorded figures for the region (e.g. the saltiest mooring observations documented within Cape Darnley approach a maximum salinity of 34.8 *psu* [5]). Therefore, the bias observed was most likely a ROMS issue.

Improvements may be obtained via a finer-scale ocean model configured to the specific study region, enabling tuning to better represent specific local processes [21, 72]. The ROMS implementation was also climatological; a more direct comparison with the observational dataset would be possible from an inter-annual ROMS implementation (e.g. with forcing that coincides with the SES data, *i*.*e*. 2007–2015). Future developments may explore a fully-coupled sea-ice component in the model (as opposed to prescribed heat and salt fluxes) to reproduce the evolution of water masses and allow an investigation of finer scale processes; and/or configure a bio-geochemical sub-model (e.g. [73]).

Despite the saline bias found within the ocean model output, for the purposes of this study regional spatial dynamics and seasonal trends were considered priority in evaluating the model’s performance. The absolute values of salinity and temperature were less important than a sufficient representation of differences between polynyas and seasonal differences within each polynya.

### Elephant seal habitat use: Observed and predicted

Examining elephant seal tracking data, combined with ocean model output to provide regional context, revealed new insights into factors influencing habitat usage within Prydz Bay. Overall, the observed and modelled habitat usage showed high residence times in the four coastal polynyas relative to the surrounding region. The most concentrated occupancy occurred within the Mackenzie and Prydz Bay polynyas, increasingly so as the season progressed to early winter.

Interestingly, the Cape Darnley polynya was visited by a relatively high number of individuals but they spent less time here overall compared to the Mackenzie and Prydz Bay polynyas. Models relating bio-physical characteristics of polynyas with seal residency time predicted this as a suitable foraging location, with concentrated seal usage especially during PM2 (May–July) in the Cape Darnley polynya. Thus, this polynya had presumably (*i*.*e*. based on models) favourable bio-physical conditions for seal foraging activity despite the lower observed time spent. Tagging location may have played some role in the observed lower rates of residency; for those individuals tagged at Davis Station (*n* = 42) Cape Darnley is certainly available/accessible (in terms of travel distance) but may be less optimal than the more proximate options of Mackenzie and Prydz Bay.

It is appropriate here to also consider the issue of temporal scale. This study examined habitat use summarised at the seasonal scale (PM1 and PM2), suitable for a first investigation. Beyond this, the heat flux predictor field we used (averaged over the entire freezing period March–October) represents relatively static information on polynya location and area, somewhat analogous to studies including ‘distance to polynya’ as a predictor field [13, 37]. This may be considered the expected spatial “ecological footprint” of polynya activity even post-activity, *i*.*e*. during summer, and contains additional information about relative intensity of activity across polynyas. The remaining physical predictor variables we used from ROMS (*e*.*g*. surface temperatures, bottom velocities etc.) all contain more dynamic information relevant at the seasonal scale. The earlier results evaluating the ROMS model clearly showed strong seasonal changes dominating the water column structure (Figs 2 and 3), but within this also finer scale temporal dynamics (S1 Appendix). The relationship between foraging behaviour and oceanographic conditions may change with the scale investigated [30].

Further investigation examining full time-series (movement and behavioural data) obtained from individual seals will no doubt prove fruitful, and may provide insight into how individual animals respond dynamically to physical processes; for example, whether individuals reside near polynya cores or edges (or vacate) during the periods of most intense ice production. The aggregation scales (spatial and temporal) used in this study may have been too coarse to detect dynamic (also potentially interannual) environmental influences on seals foraging within the region. A finer-scale investigation may also reveal why the Cape Darnley polynya had high visitation but a lower average residency time.

Foraging of SES around Antarctica has been described in deep oceanic waters [35] around the Antarctic shelf break [28] and in shelf waters. Within the greater Prydz Bay region, this study revealed the significance of bathymetry as a physical predictor for both seasonal GAMMs, describing a predominant depth for SES habitat usage between ~200–700 m. While this study did not examine open ocean foraging, those seals that migrate to this area clearly focus their time in the shelf and shelf-break vicinities, supporting the concept that the shelf region generally represents favourable foraging habitat [30, 35, 36]. Within this region, coastal polynyas have been described as key oceanographic features [1, 5, 29]; the importance of surface heat flux (a proxy for polynya area) as a predictor of habitat usage implicated polynyas as ecologically important regions for SES within the Prydz Bay region during both PM1 (February–April) and PM2 (May–July).

Residence time during PM2 showed concentrated polynya use and a reduced usage of other available shelf habitat. SES are influenced by the extent of sea-ice [74], and the majority of Prydz Bay is ice-covered during PM2. Concentrated polynya use during this season may have been due to habitat contraction because of ice formation and subsequent breathing constraints; however, the persistence of polynya usage during the previous season (PM1) suggested that there may be foraging benefits for polynya fidelity across seasons. The potential negative influence of colder waters on the mobility of prey such as fish and squid [30, 75] is a phenomenon that may be at play in the cold bottom waters of polynyas. Polynyas support high phytoplankton blooms compared to surrounding ice-covered waters in early spring and have been described as sites of concentrated biological activity supporting rich ecosystems throughout the year [7, 76]. Primary productivity (surface Chl-*a*) was represented within each seasonal predictive model as an average of the previous season to support the development of secondary production. The significance of this predictor, as well as surface heat flux and bathymetry, suggested that together polynya location and biological production were important factors determining relative rates of habitat usage within the Prydz Bay region, especially leading into winter.

In the Commonwealth Bay polynya, East Antarctica, it has been hypothesised that towards the end of summer, surface productivity is convected through the water column [68] leading to a sub-surface Chl-*a* maximum that supports secondary productivity (zooplankton, small fish etc.) used by seals later in the season. The influence of Chl*-a* within both statistical models was likely due to this relationship between high rates of primary productivity during early spring and summer and the effect this has on secondary production within polynyas. Vertical ROMS velocities (W), a product of brine rejection from sea-ice production [2–4], revealed sinking water specifically within Prydz Bay polynya and Mackenzie polynya. This vertical movement, which may have entrained primary production down through the water column, was significant in describing habitat use during PM2. A higher resolution ocean model could enable an investigation of these fine-scale water movement features to verify this transfer of biomass.

Notably, diving behaviour of SES over the Antarctic shelf (and other continental shelves) is thought to be predominantly benthic (e.g. >75% of dives) [30, 31]. With this information, an expansion to the above hypothesis [68] can be proposed, whereby the bio-physical coupling from surface to subsurface productivity is likely to extend throughout the water column as it becomes fully convected later in the season to promote pelagic-benthic coupling, a linkage between the surface pelagic system and the benthos. Recent work has highlighted the diversity of benthic community assemblages that are strongly influenced by bathymetry and other water characteristics, including distance to polynyas [37]. Through enhanced vertical carbon flux, polynyas may support rich benthic communities [77]. A productive benthic community could represent a relatively stable foraging opportunity for migratory predators, in comparison to seasonally transient pelagic production in oceanic waters.

Future research should endeavour to collect observations which would enable the validation of this hypothesis. Historical fish data suggested a greater number of species and increased biomass around regions of warm inflow, and within the Cape Darnley polynya. However, the dataset provided poor spatial coverage and there was little information for the other polynyas. The age and scarcity of this dataset highlighted the need for updated sampling of ecological communities within the Prydz Bay region. Specifically, benthic and pelagic trawl surveys of polynya and non-polynya areas within the region and ideally targeting of both inflow and outflow areas. This would further our understanding of the coupling between bottom and upper water layers, and the implications for benthic communities, and be of great value in providing a better biological description of prey availability for SES and other diving marine predators.

## Conclusion

The results of this study suggest that the most important foraging locations for juvenile male southern elephant seals within Prydz Bay region are polynyas, particularly the Cape Darnley, Mackenzie and Prydz Bay polynyas. These polynyas vary in their levels of activity, are impacted by the central gyre within the region and correspond to areas of cold water outflows and warm water inflows, respectively. Future vessel-based survey work targeting the question of whether benthic communities and associated fish assemblages are more productive inside or outside of these areas would provide valuable insights into the true nature of the proposed pelagic-benthic coupling. Obtaining prey field data at relevant spatio-temporal scales is expensive but necessary to enable a better biological understanding of how prime foraging habitat is structured, and provide a pathway into characterising the region as habitat for other marine predators such as other seals, penguins and flying seabirds.

## Supporting information

S1 Video Animation of the annual sea-surface temperature within the Prydz Bay region. (MP4)

S2 Video Animation of the annual temperature within Cape Darnley polynya transect. (MP4)

S3 Video Animation of the annual temperature within Mackenzie polynya transect. (MP4)

S4 Video Animation of the annual temperature within Prydz Bay polynya transect. (MP4)

S5 Video Animation of the annual temperature within West Ice Shelf polynya transect. (MP4)

S1 Text Regional Ocean Modelling System animation text. (DOCX)

S1 Appendix ROMS polynya time-series. (DOCX)

S2 Appendix Seal-based CTD observations within polynyas. (DOCX)

S3 Appendix Supplementary statistics supporting model development. (DOCX)
