# Supplementary material for: Bio-physical characterisation of polynyas as a key foraging habitat for juvenile male southern elephant seals (Mirounga leonina) in Prydz Bay, East Antarctica
Source: PLoS One. 2017 Sep 13;12(9):e0184536. doi: 10.1371/journal.pone.0184536 (PMC5597224; doi:10.1371/journal.pone.0184536)
Supplement: S1 Text — (DOCX) [file pone.0184536.s006.docx]

## S1 Text. Regional Ocean Modelling System animation text

Contained within this text are electronic supplementary material (provided externally). A brief discussion is detailed.

The animation of surface temperature showed apparently reasonably simulation of oceanic conditions within the greater Prydz Bay region, reproducing the main expected features. The cyclonic gyre within the centre of the bay was represented, with inflows of warmer waters at ~ 80°E and outflows of colder waters around Cape Darnley polynya (~69°E) Amery Ice Shelf (~70 - 75°E). Cape Darnley polynya and Mackenzie polynya both remained cooler throughout the year when compared to the rest of the bay. The advance of sea-ice (~ day 70) was represented by warmer surface temperatures; the simulation of ice above the ocean within ROMS acts as an insulating layer for the ocean.

These animations revealed the dynamic nature of the four polynyas within Prydz Bay. Seasonal cooling was evident during the middle of the year, with a loss of vertical stratification leading to water column homogeneity in the winter; this was especially clear within Cape Darnley polynya. Additionally, the transects indicated cold downslope flows off-shelf of waters originating within Mackenzie and Cape Darnley polynyas. This was not evident for the other two transects. On the Prydz transect warmer waters dominated the shelf break region to 66.5°S.
