# Supplementary material for: Bio-physical characterisation of polynyas as a key foraging habitat for juvenile male southern elephant seals (Mirounga leonina) in Prydz Bay, East Antarctica
Source: PLoS One. 2017 Sep 13;12(9):e0184536. doi: 10.1371/journal.pone.0184536 (PMC5597224; doi:10.1371/journal.pone.0184536)
Supplement: S1 Appendix — (DOCX) [file pone.0184536.s007.docx]

## **S1 Appendix. ROMS polynya time-series and transects**

This appendix contains supplementary transects complimenting Figure 4, in addition to temperature and salinity at depth time-series from within the centroid of each of the four Prydz Bay region polynyas illustrating the simulated annual cycle in ROMS. These figures supplement the information in available in Figures 5 and 6.


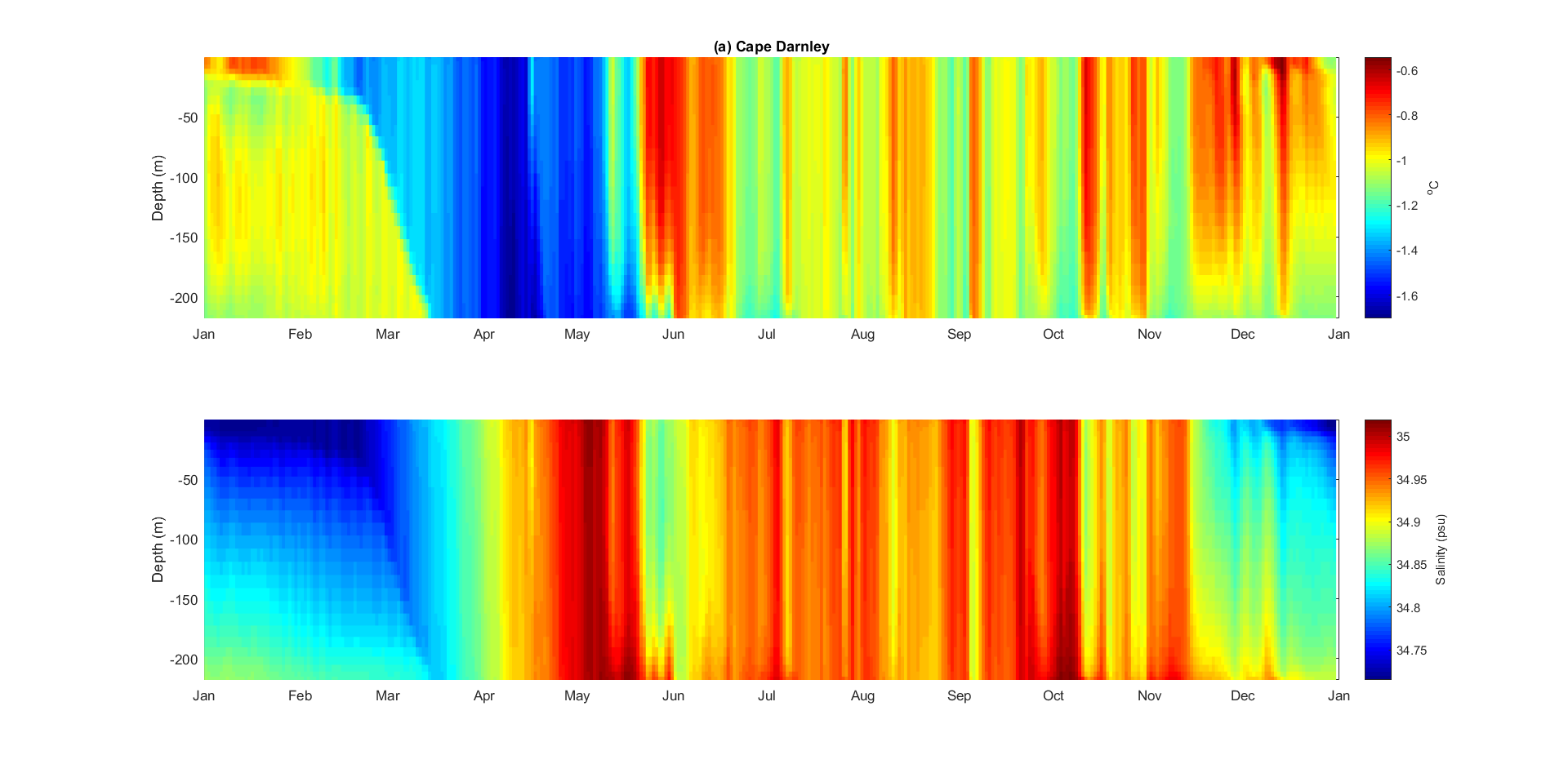

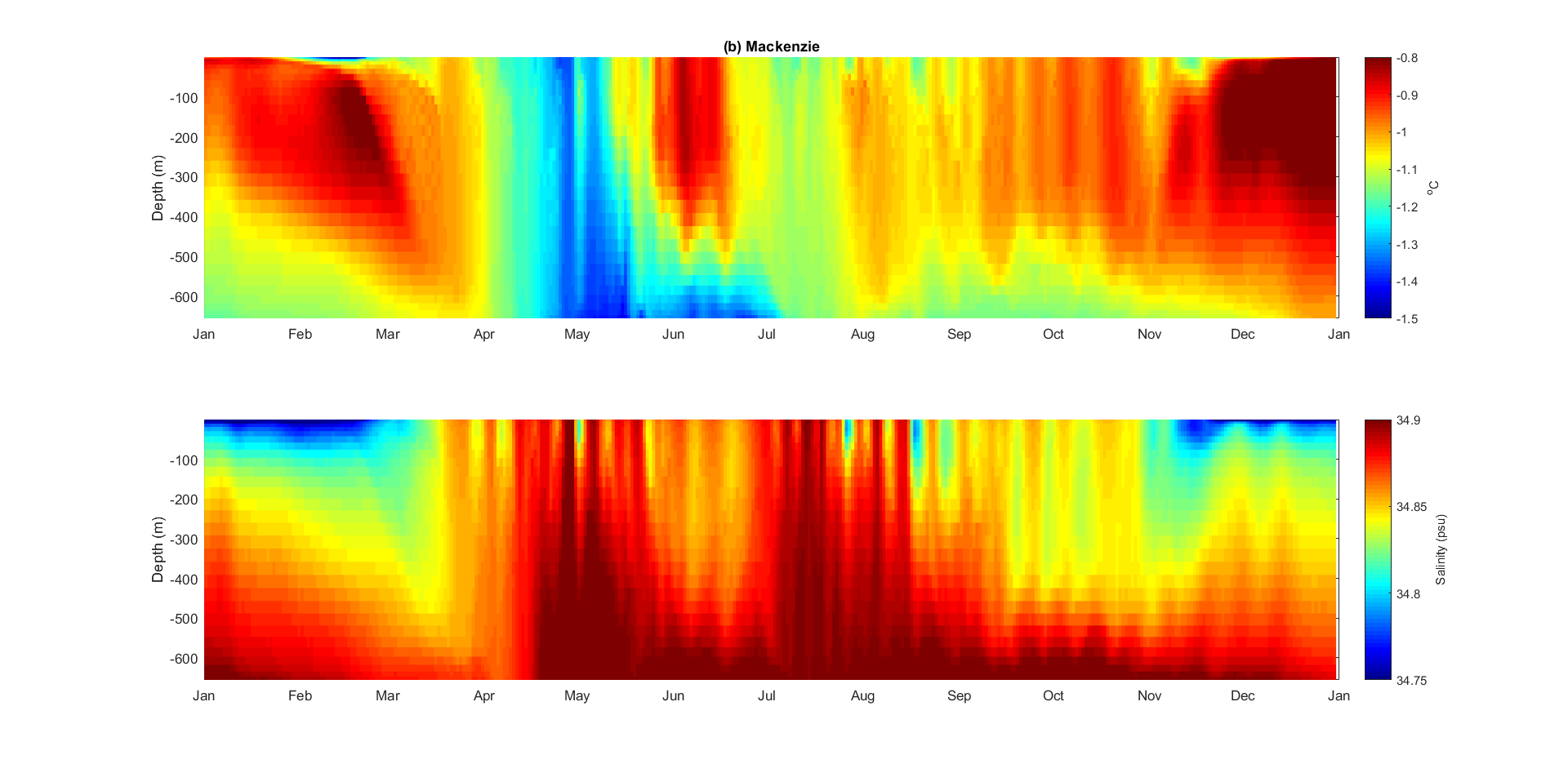

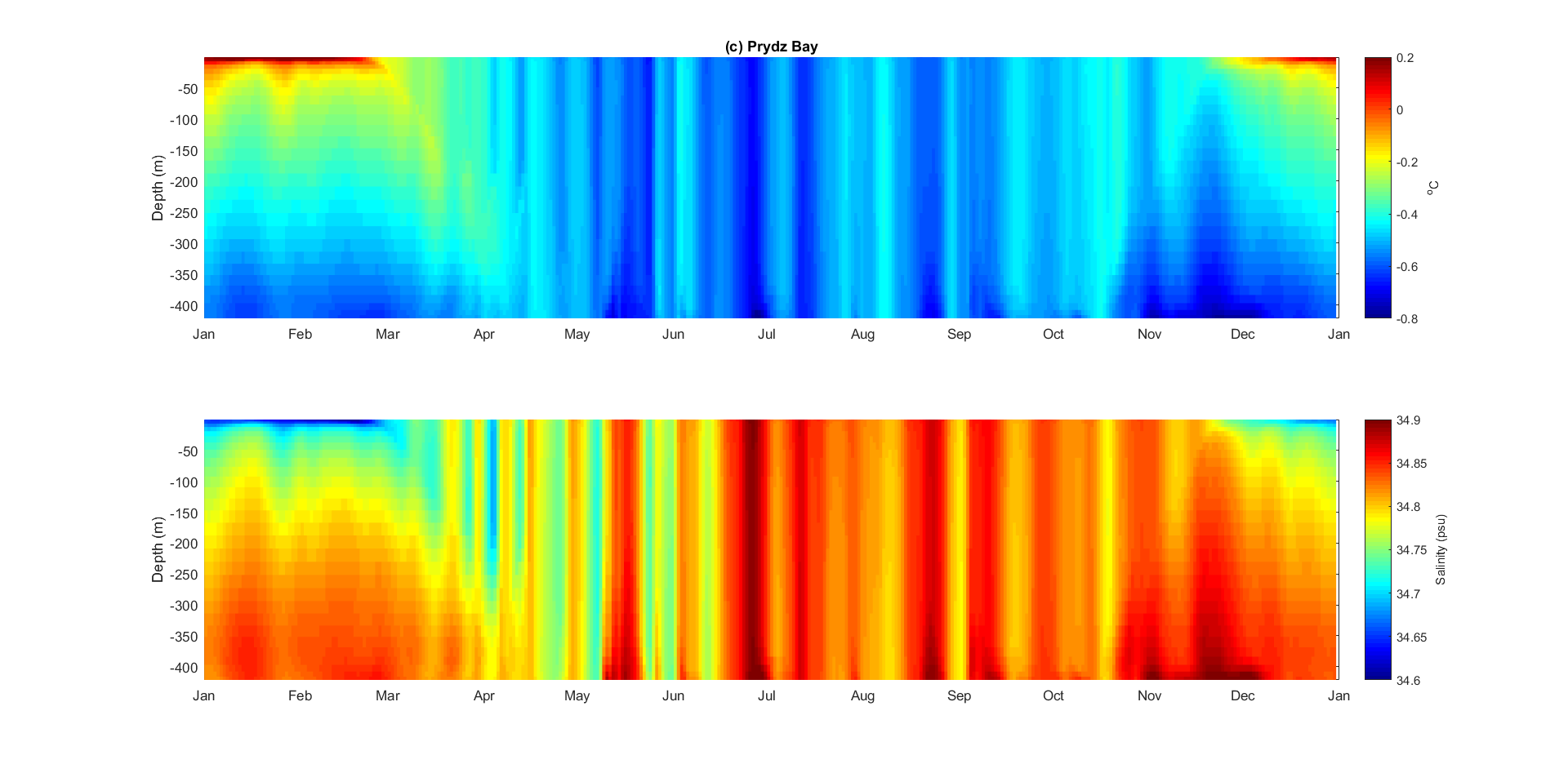

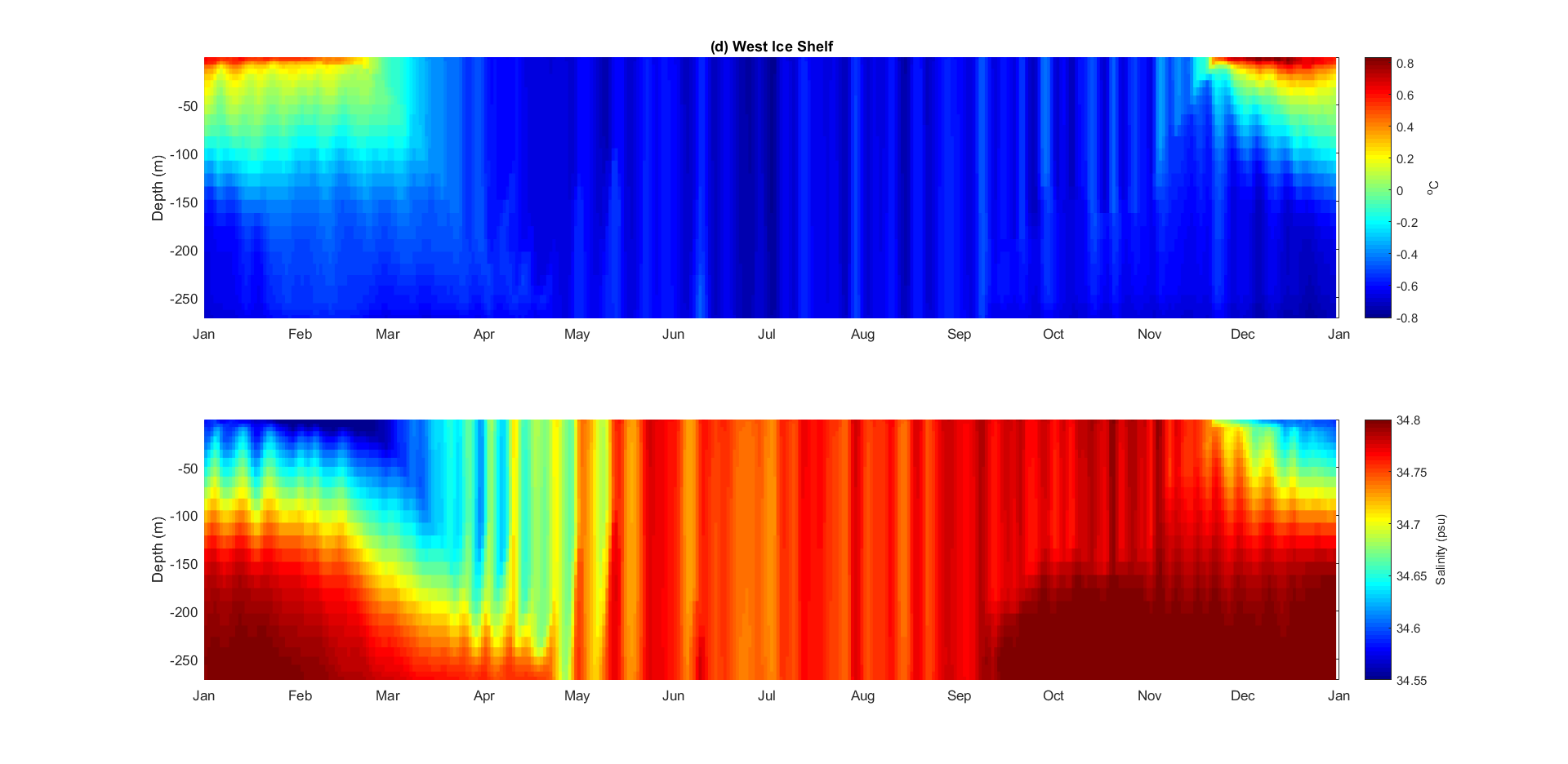


**Fig A: Annual temperature and salinity profiles within the centroid of four Prydz Bay regional polynyas;** (a) Cape Darnley, (b) Mackenzie, (c) Prydz Bay and (d) West Ice Shelf. In each case top panels show potential temperature (°C) and bottom panels show salinity (psu). Note scale bars vary between polynyas.

Warm, fresh mixed layers during summer (start and end of each panel) were evident for all polynyas. This deepened as the water column evolved to colder conditions during autumn and the impact of ice production was clearly shown throughout the freezing season. The effect of ice production on the water column became evident during mid-March. This was characterised by pulses of cold, saline water and a shift to a more heterogeneously mixed water column; these pulses generally persisted until mid-November. The same trends were observed across polynyas, although West Ice Shelf polynya seemed to remain the most heterogeneous with relatively unchanged cold, saline waters until a warming change at the end of November coinciding with the ice melt, indicating this smallest polynya to also be the weakest. West Ice Shelf was also characterised by the greatest temperature change, warming to ~ 0.8°C in summer. Overall Mackenzie polynya was the coldest, likely due to the immediate influence of the Amery ice shelf, reaching an annual temperature maximum of only – 0.8°C. This is also the deepest of the polynyas and showed very cold saline waters at depths of 600m. Cape Darnley polynya was similarly cold, with an annual maximum of – 0.6°C, and represented the most saline waters.


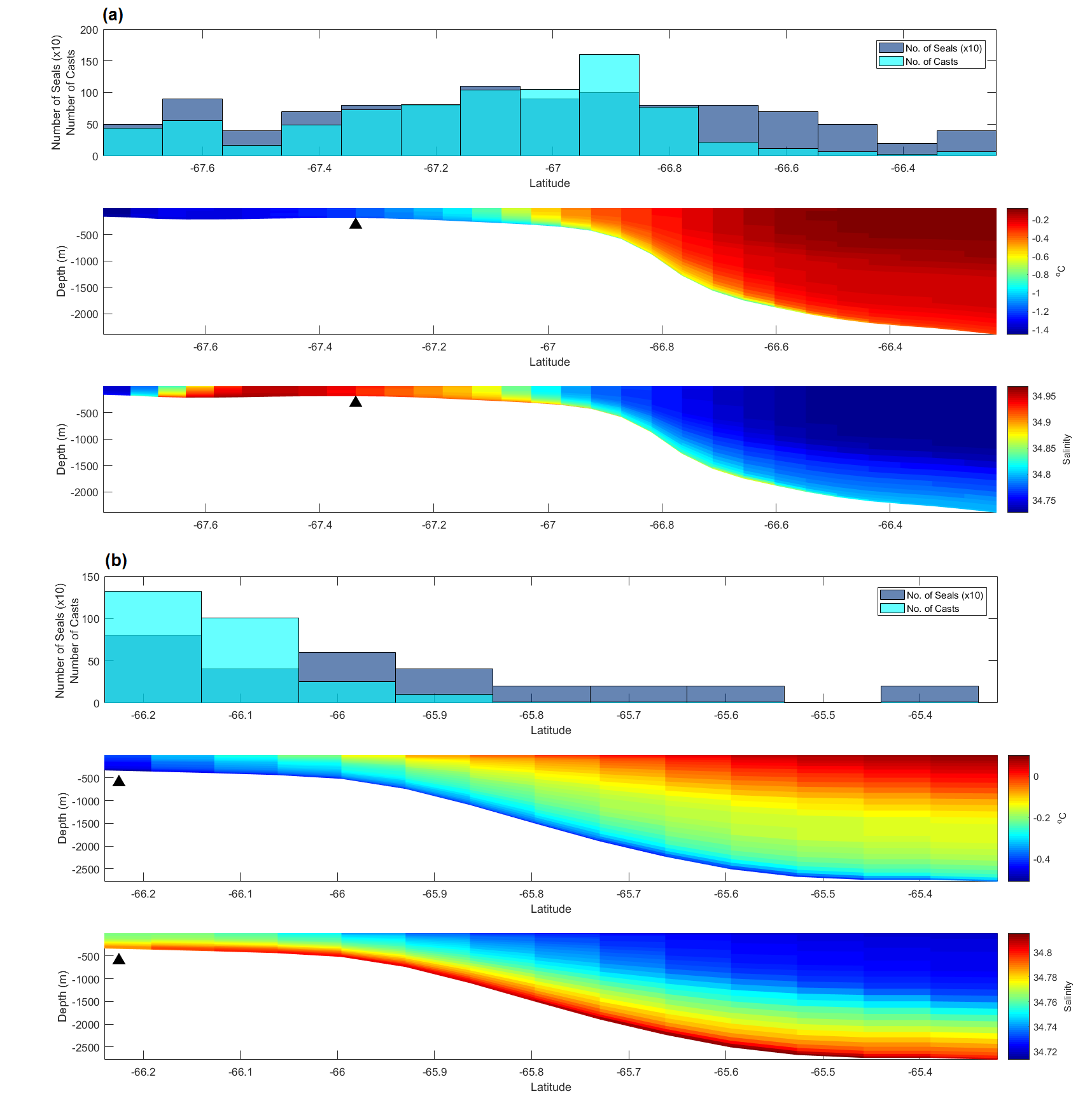


**Fig B. Virtual transects showing ROMS temperature and salinity model output in relation to the number of observed seals and seal CTD casts.**

Virtual transects ran north-south from two polynya centres to the shelf break. Modelled temperature and salinity was averaged over the freezing period (March – October). Transects represent a) Cape Darnley and b) West Ice Shelf polynyas. The number of seals in the top panel for each figure was multiplied by a factor of 10 for improved visualisation. Polynya centroid location represented by a black triangle.
