# Supplementary material for: Bio-physical characterisation of polynyas as a key foraging habitat for juvenile male southern elephant seals (Mirounga leonina) in Prydz Bay, East Antarctica
Source: PLoS One. 2017 Sep 13;12(9):e0184536. doi: 10.1371/journal.pone.0184536 (PMC5597224; doi:10.1371/journal.pone.0184536)
Supplement: S2 Appendix — (DOCX) [file pone.0184536.s008.docx]

##
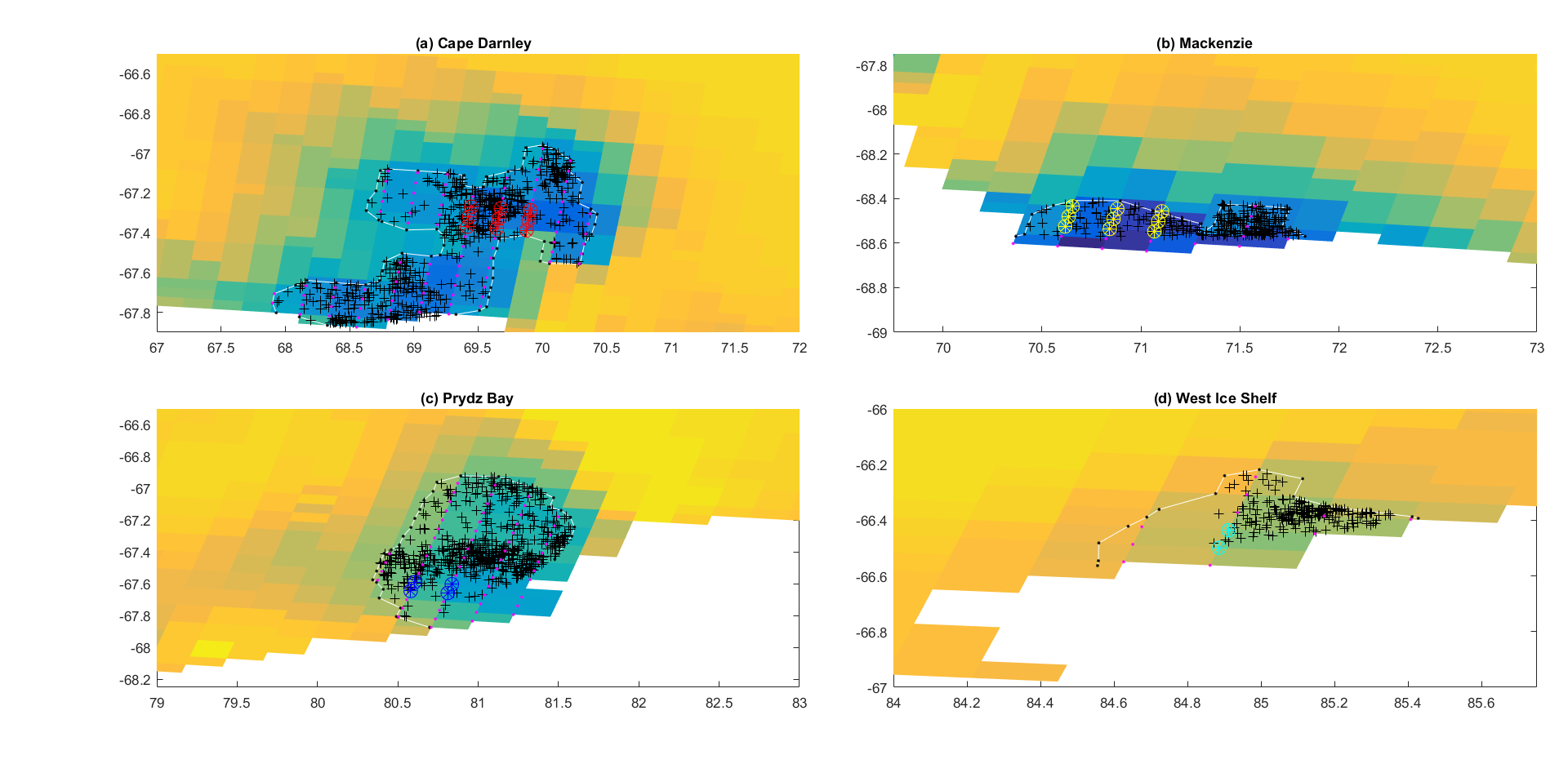
S2 Appendix. Seal-based CTD observations within polynyas

**Figure A: Maps showing SES CTD cast locations within (a) Cape Darnley, (b) Mackenzie, (c) Prydz Bay and (d) West Ice Shelf polynyas.** Background shows average heat flux throughout the freezing season (March – October). The heat flux threshold used as the polynya boundary is depicted by a white contour. Black + symbols represent the location of each unique cast, and the ROMS grid cells used to represent centroids are shown as open coloured circles within each polynya (Red, yellow, blue and cyan respectively).


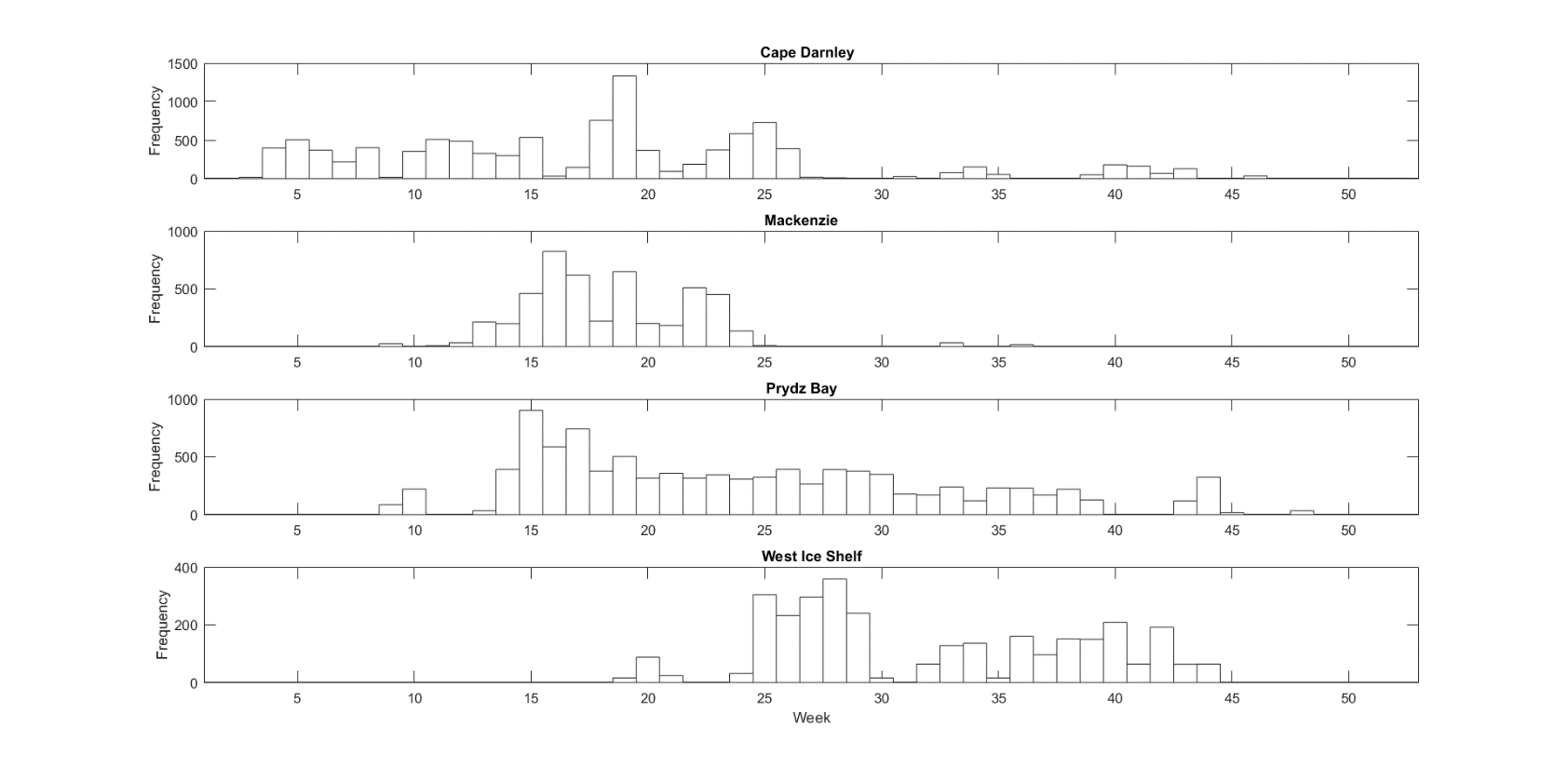


**Figure B: Number of casts recorded within each polynya for each week of the year.** Cape Darnley polynya, Mackenzie polynya, Prydz Bay polynya and West Ice Shelf polynya are shown.


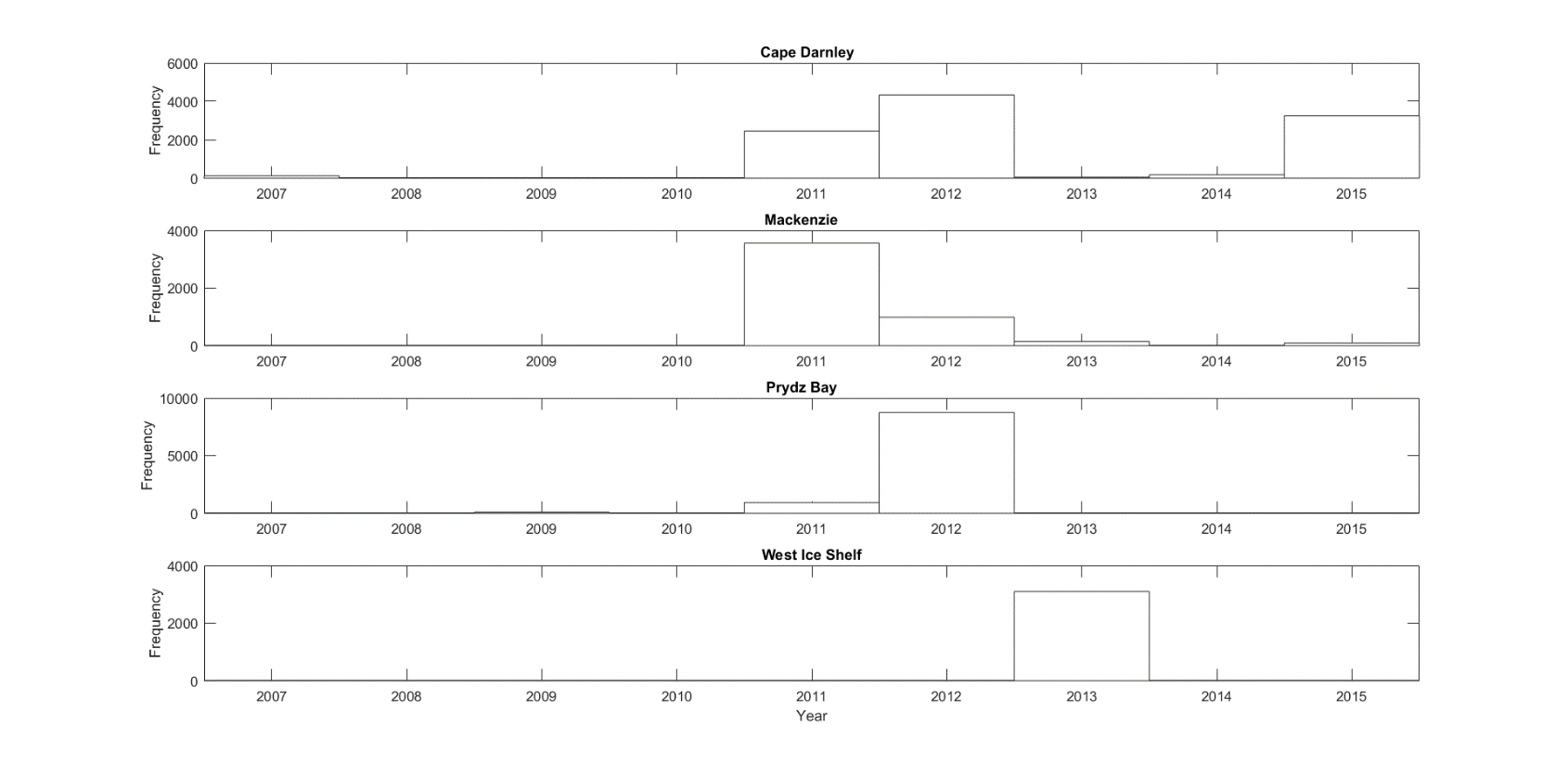


**Figure C: Number of casts recorded within each polynya for each year from 2007 – 2015.** Cape Darnley polynya, Mackenzie polynya, Prydz Bay polynya and West Ice Shelf polynya are shown.

Seal CTD observations were available for different periods across the polynyas (Figure S2.2). Mackenzie, Cape Darnley and Prydz Bay were occupied mainly during week 15 – 25 (April – June), whereas observations for West Ice Shelf spanned week 25 – 30 (mid June – July). This suggested that during winter there were a few elephant seals with strong fidelity to West Ice Shelf. The period between 2011 and 2013 represents the greatest data coverage (Figure S2.3) across the four polynyas.
