# Supplementary material for: Bio-physical characterisation of polynyas as a key foraging habitat for juvenile male southern elephant seals (Mirounga leonina) in Prydz Bay, East Antarctica
Source: PLoS One. 2017 Sep 13;12(9):e0184536. doi: 10.1371/journal.pone.0184536 (PMC5597224; doi:10.1371/journal.pone.0184536)
Supplement: S3 Appendix — (DOCX) [file pone.0184536.s009.docx]

## S3 Appendix. Supplementary statistics supporting model development

This appendix contains the statistical output and predictor layers used to build both seasonal GAMS and GAMMs.


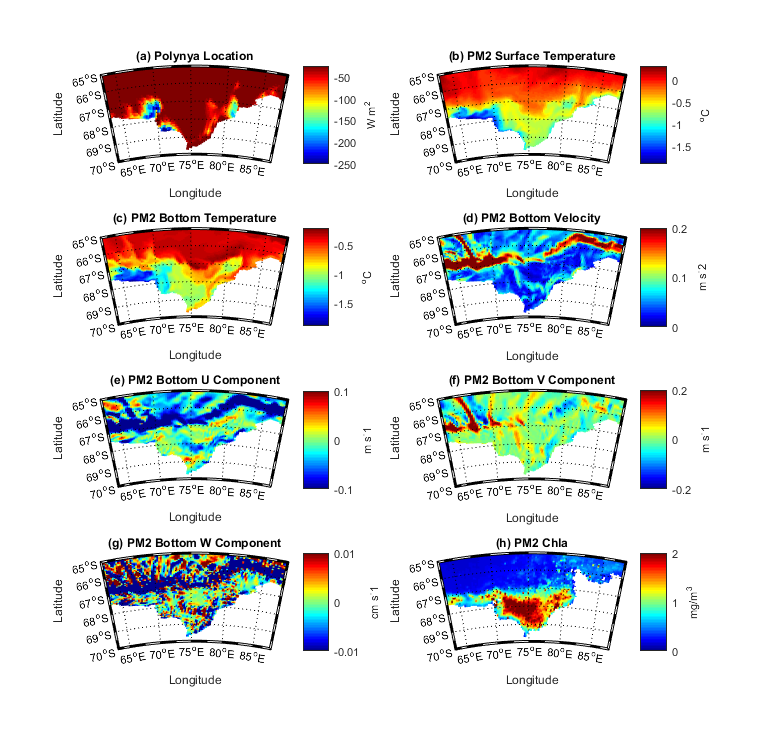


**Figure A: Physical and biological predictors fields used to build the seasonal GAMM for PM2**. a) Polynya location, surface heat flux averaged over the freezing season (March to October), b) surface temperature, c) bottom temperature, d) bottom velocity, e) bottom U component of velocity, f) bottom V component of velocity, g) bottom W component of velocity and h) surface chlorophyll averaged over the previous season (November to January). Not shown is the 9^th^ predictor field, bathymetry.

**Table A: Variance inflation factors for 7 bio-biophysical predictors used to develop a spatially correlated GAMM for PM1.** Predictors that showed collinearity were removed, and this table demonstrates a VIF less than 3 for each variable.

|  | **GVIF** |
| --- | --- |
| **heat** | 1.890001 |
| **s_temp** | 2.763258 |
| **b_temp** | 2.761394 |
| **Vel** | 1.580411 |
| **V** | 1.277488 |
| **log.bath** | 1.866736 |
| **log.chlo** | 1.773368 |

**Table B: Statistical results from developmental single-term GAMs for PM1 used to investigate the capability of bio-physical predictors at predicting elephant seal habitat use.** Adjusted R-squared values, percentage deviance explained and Akaike’s Information Criterion (AIC) are shown for 7 significant, non-correlated predictors.

|  | **R-sq.(adj)** | **Deviance Explained** | **AIC** |
| --- | --- | --- | --- |
| **S(heat)** | 0.125 | 12.8 | 5223.11 |
| **S(s_temp)** | 0.0725 | 7.46 | 5422.232 |
| **S(b_temp)** | 0.181 | 18.3 | 5001.159 |
| **S(vel)** | 0.14 | 14.2 | 5167.523 |
| **S(V)** | 0.0872 | 8.96 | 5369.034 |
| **S(log.bath)** | 0.284 | 28.6 | 4540.483 |
| **S(log.chlo)** | 0.188 | 19 | 4969.957 |

**Table C: Variance inflation factors for 7 bio-biophysical predictors used to develop a spatially correlated GAMM for PM2.** Predictors that showed collinearity were removed, and this table demonstrates a VIF less than 3 for each variable.

|  | **GVIF** |
| --- | --- |
| **heat** | 1.722681 |
| **b_temp** | 2.703924 |
| **Vel** | 2.530242 |
| **V** | 1.404855 |
| **log.bath** | 2.522258 |
| **log.chlo** | 2.633811 |
| **W** | 2.793348 |

**Table D: Statistical results from developmental single-term GAMs for PM2 used to investigate the capability of bio-physical predictors at predicting elephant seal habitat use.** Adjusted R-squared values, percentage deviance explained and Akaike’s Information Criterion (AIC) are shown for 7 significant, non-correlated predictors.

|  | **R-sq.(adj)** | **Deviance Explained** | **AIC** |
| --- | --- | --- | --- |
| **S(heat)** | 0.389 | 39.1 | 4935.422 |
| **S(b_temp)** | 0.24 | 24.3 | 5468.503 |
| **S(vel)** | 0.00147 | 0.187 | 6129.342 |
| **S(V)** | 0.0192 | 2.27 | 6093.276 |
| **S(W)** | -7.54e-05 | 0.0513 | 6133.556 |
| **S(log.bath)** | 0.315 | 31.8 | 5213.551 |
| **S(log.chlo)** | 0.281 | 28.3 | 5333.547 |


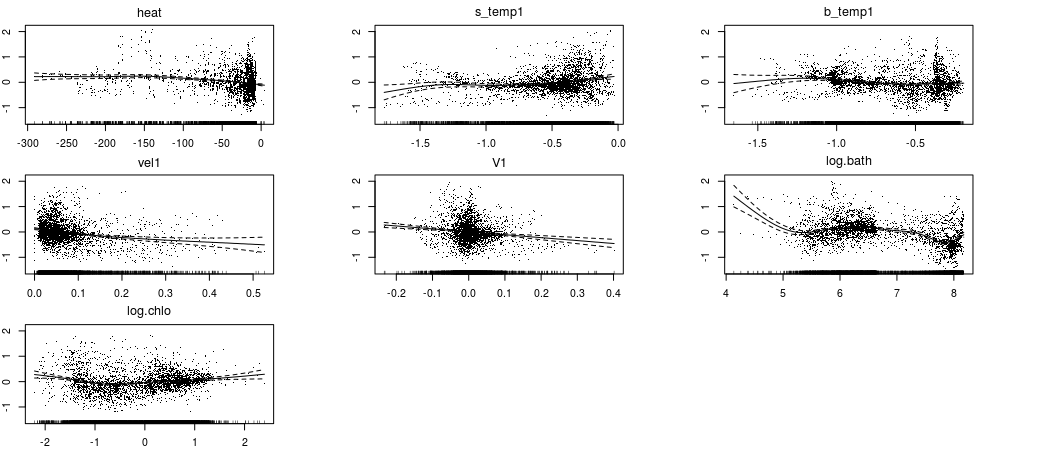

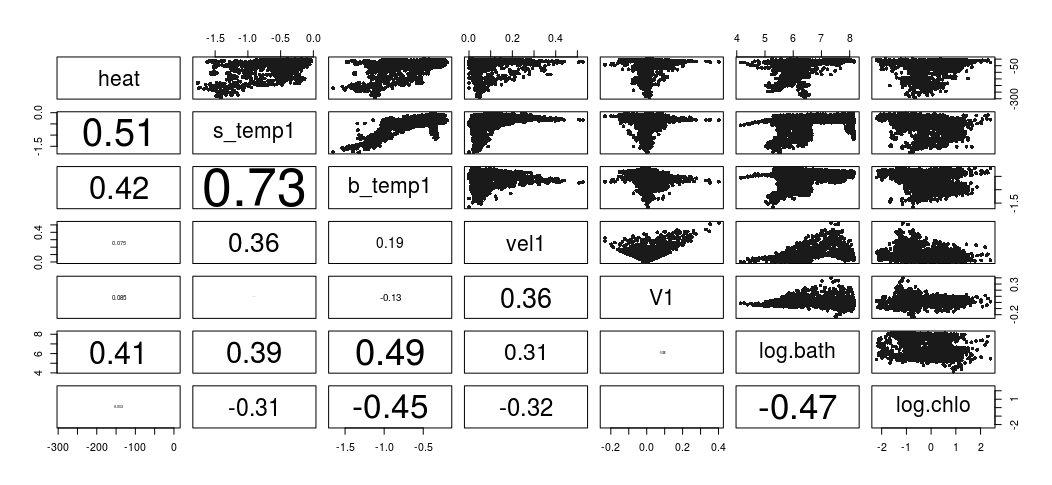
**Figure B: Correlations between predictor variables used to build a GAMM for PM1.** Correlation values are less than 0.8. Predictors shown are the ones included in the final GAMM; highly correlated predictors were previously removed.

**Figure C: Partial residual plots for significant predictors from PM1 GAMM.**


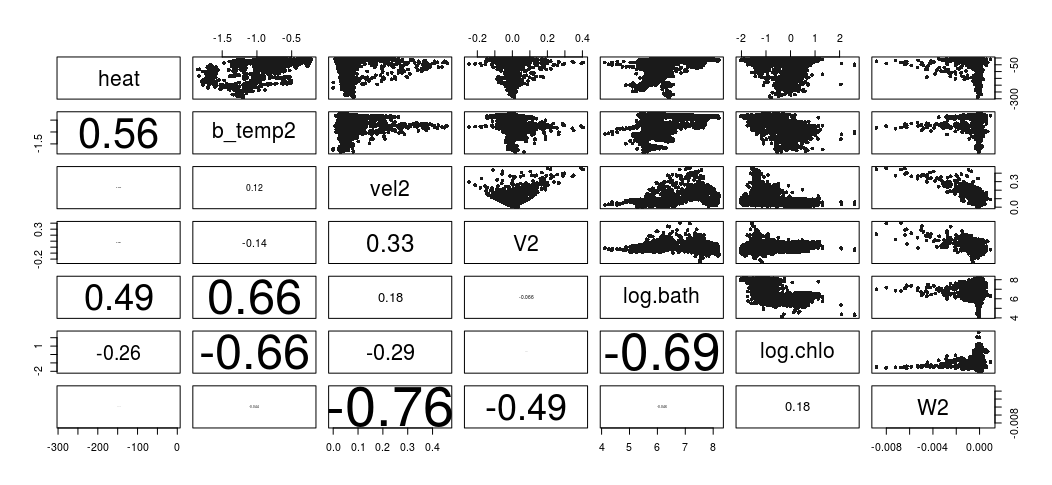
**Figure D: Correlations between predictor variables used to build a GAMM for PM2.** Correlation values are less than 0.8. Predictors shown are the ones included in the final GAMM; highly correlated predictors were previously removed.


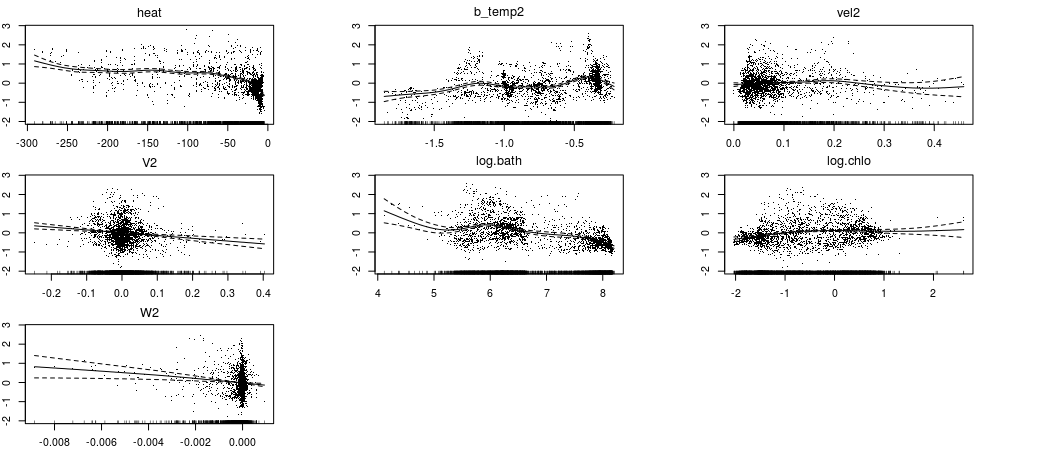


**Figure E: Partial residual plots for significant predictors from PM2 GAMM.**
